# A subpopulation of arenavirus nucleoprotein localizes to mitochondria

**DOI:** 10.1038/s41598-021-99887-5

**Published:** 2021-10-26

**Authors:** Francesca Baggio, Udo Hetzel, Lisbeth Nufer, Anja Kipar, Jussi Hepojoki

**Affiliations:** 1 Institute of Veterinary Pathology, Vetsuisse Faculty, University of Zurich, 8057 Zurich, Switzerland; 2 Center for Clinical Studies, Vetsuisse Faculty, University of Zurich, 8057 Zurich, Switzerland; 3 Department of Veterinary Biosciences, Faculty of Veterinary Medicine, University of Helsinki, 00014 Helsinki, Finland; 4 Department of Virology, Medicum, Faculty of Medicine, University of Helsinki, 00290 Helsinki, Finland

**Keywords:** Mitochondria, Arenaviruses

## Abstract

Viruses need cells for their replication and, therefore, ways to hijack cellular functions. Mitochondria play fundamental roles within the cell in metabolism, immunity and regulation of homeostasis due to which some viruses aim to alter mitochondrial functions. Herein we show that the nucleoprotein (NP) of arenaviruses enters the mitochondria of infected cells, affecting the mitochondrial morphology. Reptarenaviruses cause boid inclusion body disease (BIBD) that is characterized, especially in boas, by the formation of cytoplasmic inclusion bodies (IBs) comprising reptarenavirus NP within the infected cells. We initiated this study after observing electron-dense material reminiscent of IBs within the mitochondria of reptarenavirus infected boid cell cultures in an ultrastructural study. We employed immuno-electron microscopy to confirm that the mitochondrial inclusions indeed contain reptarenavirus NP. Mutations to a putative N-terminal mitochondrial targeting signal (MTS), identified via software predictions in both mamm- and reptarenavirus NPs, did not affect the mitochondrial localization of NP, suggesting that it occurs independently of MTS. In support of MTS-independent translocation, we did not detect cleavage of the putative MTSs of arenavirus NPs in reptilian or mammalian cells. Furthermore, in vitro translated NPs could not enter isolated mitochondria, suggesting that the translocation requires cellular factors or conditions. Our findings suggest that MTS-independent mitochondrial translocation of NP is a shared feature among arenaviruses. We speculate that by targeting the mitochondria arenaviruses aim to alter mitochondrial metabolism and homeostasis or affect the cellular defense.

## Introduction

Viruses are obligate cellular parasites and have thus adopted strategies to either promote or inhibit host cell functions for their own benefit. To hijack the host cell, viruses can target specific subcellular structures or can affect host protein levels and/or their trafficking^1^. To counteract the host defense, viruses can interfere with the functions of cellular compartments that contribute to antiviral signalling, such as mitochondria, peroxisomes, endoplasmic reticulum (ER), lipid droplets and the nucleus^1,2^. Mitochondria play an essential role in cell metabolism, but also regulate apoptosis^3^ and control immune and inflammatory responses^4^. Viruses can perturb mitochondria in several ways, e.g. by altering metabolic pathways or by inducing their fusion or fission, either to promote mitochondrial autophagy (mitophagy) or to affect the distribution of the mitochondria within the cell^5^.

The family *Arenaviridae* comprises four genera, *Mammarenavirus*, *Reptarenavirus*, *Hartmanivirus* and *Antennavirus*^6^. Arenaviruses have a single-stranded bisegmented RNA genome, except for antennaviruses that have a trisegmented genome^7^. The large (L) segment of mamm- and reptarenaviruses encodes the Z protein (ZP) and the RNA-dependent RNA polymerase (RdRp), whereas the small (S) segment encodes the glycoprotein precursor (GPC) and the nucleoprotein (NP)^7^. Hartmaniviruses and, likely, antennaviruses, lack ZP^8^. In infected cells, NP is the most abundantly expressed arenaviral protein because it has multiple roles: (1) binding to and encapsidating the viral RNA genome; (2) viral ribonucleoprotein (vRNP) complex formation; (3) interaction with ZP in assembly and budding^9^; and (4) host immunosuppression via the inhibition of interferon (IFN) signaling^9–16^.

Reptarenaviruses were discovered in the early 2010s in captive constrictor snakes with boid inclusion body disease (BIBD)^17–19^. BIBD manifests by the formation of electron-dense cytoplasmic inclusion bodies (IBs) in most cell types of affected snakes^20,21^. The IBs, comprising mainly reptarenavirus NP^17,19,22^, appear not to be cytopathic^19,23^. However, snakes with BIBD often die from secondary bacterial, fungal or protozoal infections, or neoplastic processes^24,25^. We, as the BIBD group, have isolated several reptarenaviruses including University of Helsinki virus 1 (UHV-1) and University of Giessen virus 1 (UGV-1), from *Boa constrictor* snakes with BIBD^19,26^. We have furthermore shown that UHV-1 can also infect mammalian cells (Vero E6) at suitable growth temperatures, resulting in IB formation^27^. UGV-1, on the other hand, is the reptarenavirus whose S segment (referred to as S6 by Stenglein and collaborators) is most often identified in snakes with BIBD^28,29^. UGV-1 also grows to high titers in our cell culture models and produces the characteristic IBs in infected cells^8^. Also, we have developed specific detection tools including constructs bearing UHV-1 and UGV-1 NP and GPC sequences and several antisera (broadly cross-reactive among reptarenavirus species) against these viruses^19,27,30^.

The mechanisms of IB formation during reptarenavirus infection are not known in detail. While performing an ultrastructural study on reptarenavirus infected boid cells, we observed an accumulation of electron-dense material reminiscent of IBs within mitochondria, accompanied by mitochondrial deformation. These observations led us to study the mitochondria-associated inclusions and revealed that a fraction of reptarenavirus NP enters the mitochondria during infection and transfection. By combining ultrastructural imaging and molecular biology techniques, we confirm that reptarenavirus NP indeed enters and induces IB formation in the mitochondria both in vitro and in vivo. Our results further suggest that mitochondrial localization of NP is a common feature among viruses of different arenavirus genera, indicating an evolutionarily conserved function that remains to be identified.

## Results

### Reptarenavirus NP forms inclusions within mitochondria in vitro and in snakes with BIBD

**Figure 1:**
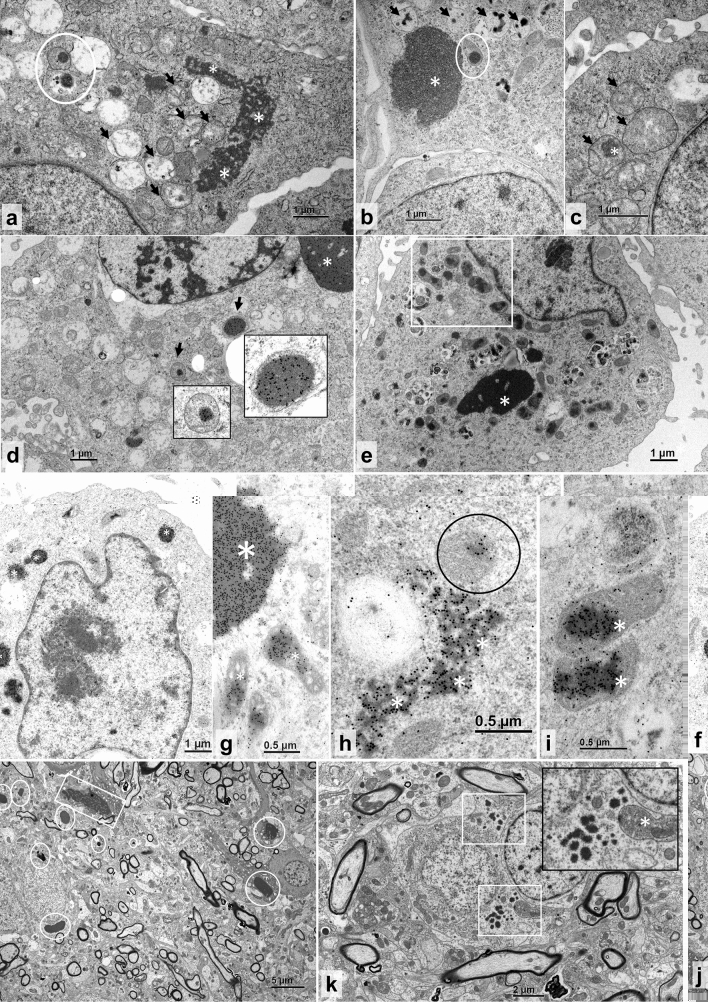
TEM and immuno-EM of reptarenavirus-infected and NP-transfected boid cells, and TEM of a BIBD-positive *B. constrictor* brain. (**a–c**) TEM, permanent cell culture derived from *B. constrictor* kidney (I/1Ki), infected with UGV-1, at three dpi. (**a**) Large irregular cytoplasmic inclusion body (IB; asterisks), vacuolated mitochondria (arrows) and partly ruptured mitochondrion with electron-dense IBs in the matrix (circle). (**b**) Large electron-dense cytoplasmic IB (asterisk), one mitochondrion with IB in the matrix (circle), and several vacuolar structures consistent with vacuolated mitochondria with small IBs (arrows). (**c**) Swollen mitochondria with finely granular disintegrated matrix (arrows) and one IB (asterisk). (**d**) Immunogold labelling of the reptarenaviral NP, I/1Ki infected with UGV-1, at three dpi. Large NP-positive cytoplasmic IB (asterisk) and several NP-positive IBs within the matrix of mitochondria (arrows). Inserts: higher magnification of the areas indicated by the arrows. (**e–i**) I/1Ki transfected with UHV1-NP-FLAG, at three dpt. (**e**) TEM, cell with large electron-dense IB (asterisk) and multiple mitochondria with IB formation (highlighted by a white rectangle). (**f–i**) Immunogold labelling of reptarenaviral NP. (**f**) Positive reaction in small electron-dense cytoplasmic IBs (asterisks). (**g**) Large electron-dense IB with positive reaction (larger asterisk) and individual mitochondria with positive IBs within the matrix (smaller asterisks). (**h**) Irregular shaped, more electron-lucent, presumably earlier cytoplasmic IB (asterisk) and single mitochondrion with positive reaction within the matrix (circle). (**i**) Mitochondria with positive IBs within the matrix (asterisks) at higher magnification. (**j–k**) TEM, neurons. (**j**) Numerous < 1–3.5 µm sized, round, smooth edged electron-dense cytoplasmic IBs (circles) and one larger, less electron-dense, irregularly shaped IB with coarser margins (square). (**k**) Neurons with small, 0.1–3 µm sized electron-dense cytoplasmic IBs (white squares). Insert: higher magnification depicting irregularly shaped IB borders and a swollen mitochondrion with disorganized coarse electron-dense matrix (asterisk).

In the attempt to identify any subtle cytopathic effects of reptarenaviruses, we investigated the boid kidney (I/1Ki) cell line infected with UGV-1 by transmission electron microscopy (TEM). At 3 days post-infection (dpi) reptarenavirus infected cells exhibited the characteristic electron-dense round to oval IBs with smooth boundaries^19^ (white circles, Fig. 1a,b), but also less electron-dense IBs with rough edges (white asterisks, Fig. 1a–c). The mitochondria exhibited pronounced vacuolization and disruption of the matrix with loss of cristae structures (black arrows, Fig. 1a–c) and occasional evidence of intramitochondrial electron-dense structures resembling reptarenavirus-induced IBs (white circles, Fig. 1a,b; white asterisk, Fig. 1c). We identified the swollen and inclusion-containing structures as mitochondria based on their morphological features, with outer membrane, matrix and cristae, and density characteristics. To study if the electron-dense material within mitochondria represents reptarenavirus NP, we performed immuno-electron microscopy (immuno-EM). Immunogold labeling of UGV-1 infected I/1Ki cells with anti-reptarenavirus NP antibody at three dpi confirmed that the material within mitochondria is indeed NP (black arrows, Fig. 1d). To study if reptarenavirus NP enters the mitochondria in the absence of other viral proteins, we transfected I/1Ki cells with a construct bearing FLAG-tagged UHV-1 NP (UHV1-NP-FLAG). At 3 days post-transfection (dpt), UHV1-NP-FLAG transfected I/1Ki cells displayed electron-dense and electron-lucent (asterisks, Fig. 1e,f,h; bigger asterisk Fig. 1g) as well as smaller mitochondrial IBs (white rectangle, Fig. 1e; smaller asterisks, Fig. 1g; black circle, Fig. 1h; asterisks, Fig. 1i), as detected by both TEM (Fig. 1e) and immuno-EM (Fig. 1f–i), reflecting the observations made in infected cells (Fig. 1a–d). Images of mock infected/transfected controls are provided (Supplementary Fig. S2a–c).

With the aim to determine whether the mitochondrial IB/NP localization observed in vitro would also occur in vivo, we examined the brain of a *Boa constrictor* snake with confirmed BIBD. Both the electron-dense, variably sized, round IBs with a smooth outline and the irregularly shaped, less electron-dense IBs with irregular borders that we saw in vitro (Fig. 1a–i) were present in neurons (white circles and squares, Fig. 1j,k; white asterisks and white circle, Supplementary Fig. S1a–d). Mock-infected controls from the brain-derived V/4Br cell line are provided (Supplementary Fig. S2d–g). Mitochondria that exhibited the less electron-dense IBs were often swollen, partly vacuolated and disrupted, with a granular, electron-dense matrix and indistinct, possibly ruptured outer membrane (white asterisk, Fig. 1k; white rectangle, black arrowheads and black arrows, Supplementary Fig. S1a, b). Both types of IBs as well as occasional mitochondria contained viral NP, as shown by immuno-EM (white asterisks, white circle and black arrows, Supplementary Fig. S1c,d), suggesting that the phenomenon occurs during natural infection.

### Identification of an in silico predicted mitochondrial targeting signal (MTS) and generation of MTS-mutant NPs

**Figure 2:**
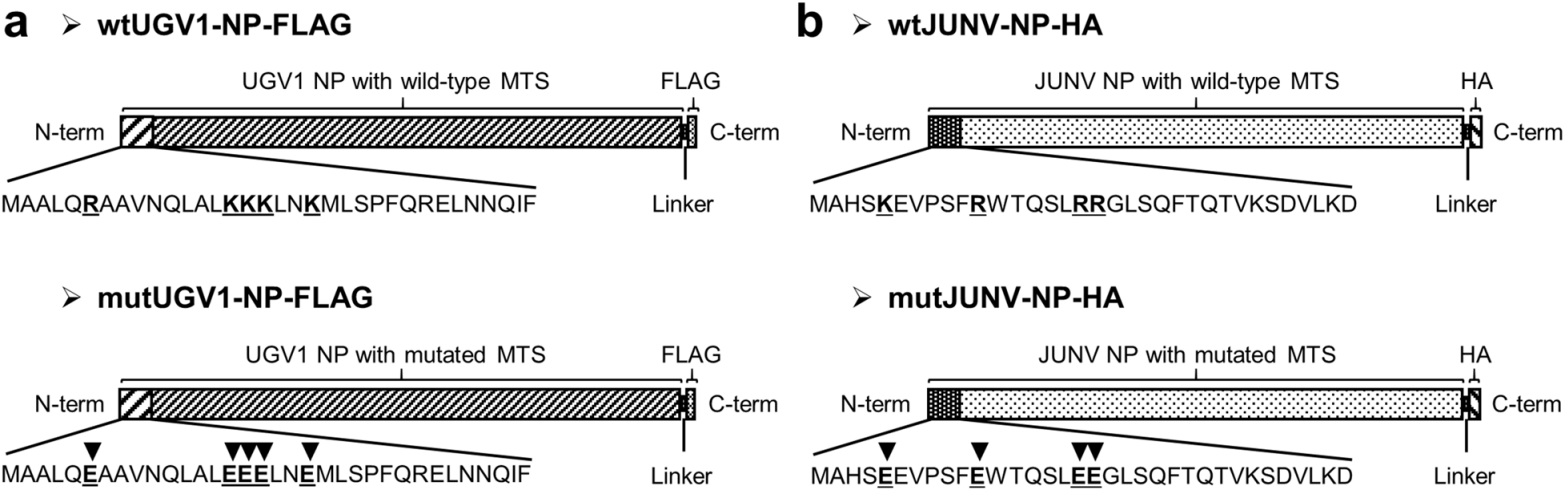
Arenaviral NPs transfection constructs. (**a**,**b**) Schematic representation of the wild-type (wt) and the mutated (mut) versions of the UGV-1 NP (**a**) and JUNV NP (**b**) expressed from a pCAGGS-FLAG or pCAGGS-HA construct, respectively, and used for transfections. Black arrowheads in the putative MTSs (first N-terminal 34 amino acids shown) indicate the positions of the amino acid substitutions of the mut versions compared to the corresponding wt sequences. Both wt and mut UGV-1 NPs are fused in frame with a C-terminal FLAG tag, separated by a linker sequence (**a**). Both wt and mut JUNV NPs are fused in frame with a C-terminal HA tag, separated by a linker sequence (**b**).

Many newly synthesized proteins destined to the mitochondria carry an MTS, an N-terminal positively charged amphipathic helix of 15–70 amino acid residues^31^. In silico analyses based on two online prediction tools, Target P 1.1 (http://www.cbs.dtu.dk/services/TargetP-1.1/index.php) and Mitoprot II (https://ihg.gsf.de/ihg/mitoprot.html) revealed a putative MTS in all tested reptarenavirus NPs (Supplementary Table S1). We then studied whether the NPs of viruses from the other genera of the family *Arenaviridae* would produce similar predictions. Indeed, many mammarenavirus NPs contain a putative MTS, however, neither hartmani- nor antennavirus NPs contain an MTS by prediction (Supplementary Table S1). In order to elucidate whether the predicted MTS is responsible for the import of NP into mitochondria, we generated recombinant expression constructs in which the positively charged interface in the NP N-terminus is replaced by negative charges. We used Jpred, a protein secondary structure prediction tool^32^, to confirm that the introduced mutations do not alter the helical structure of the putative MTS. Target P 1.1 and Mitoprot II served to demonstrate a loss of mitochondrial translocation potential of the NPs with mutated MTS (Supplementary Table S1). MTS mutations were introduced to the NPs of UGV-1 and Junin virus (JUNV), to generate FLAG- and HA-tagged constructs: wild-type (wt) UGV1-NP-FLAG, mutated (mut) UGV1-NP-FLAG, wtJUNV-NP-HA and mutJUNV-NP-HA (Fig. 2a,b). UHV-1, Lymphocytic choriomeningitis virus (LCMV) and Haartman Institute snake virus 1 (HISV-1) NP constructs, UHV1-NP-FLAG, LCMV-NP-HA and HISV1-NP-FLAG, served as controls.

### Mitochondrial translocation of NP is independent of the in silico predicted MTS

**Figure 3:**
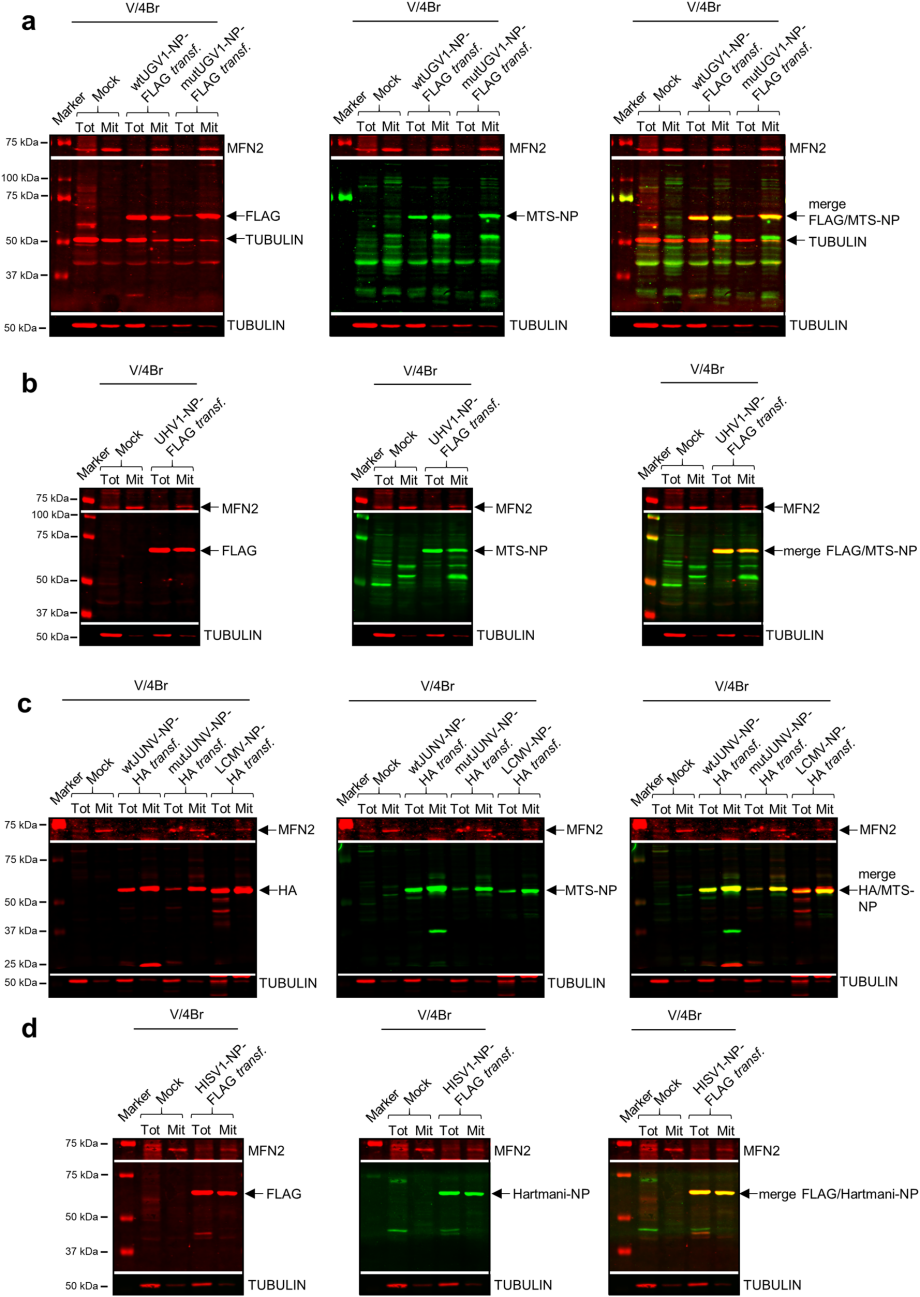
Immunoblotting studies on boid cells transfected with different arenaviral NPs. (**a–d**) Immunoblotting analyses of whole-cell lysates (Tot) and mitochondrial preparations (Mit) obtained from *Boa constrictor* V/4Br cells transfected with constructs expressing wt or mutUGV1-NP-FLAG (**a**), UHV1-NP-FLAG (**b**), wt or mutJUNV-NP-HA, or LCMV-NP-HA (**c**) and HISV1-NP-FLAG (**d**), all at three dpt. Non-transfected (Mock) samples were used as negative controls. About 1/5 of the transfected cells were used for whole-cell lysate procedure (Tot) and the other 4/5 for mitochondria isolation (Mit). 20 µg (**a**,**b**) or 12 µg (**c**,**d**) of protein per sample from both Tot and Mit were loaded on standard SDS-PAGE gels followed by immunoblotting analyses. The nitrocellulose membranes were incubated sequentially with the following antibodies in the presented order: (1) mouse anti-FLAG tag 1:500 (**a**,**b**,**d**) or mouse anti-HA tag 1:500 (**c**); (2) rabbit anti-MTS-NP 1:200 (**a**–**c**), or rabbit anti-Hartmani-NP 1:500 (**d**); (3) mouse anti-tubulin 1:500 (**a–d**); (4) mouse anti-MFN2 1:200 (**a**,**b**,**d**) or 1:100 (**c**). Anti-tubulin and anti-MFN2 specific signals at known molecular weight did not require membrane stripping, except for panel c, where a stripping step was introduced before probing with mouse anti-MFN2. Tubulin and MFN2 (both in red, secondary antibody: IRDye 680RD Donkey anti-mouse) were used as cytosolic and mitochondrial marker, respectively. Arenavirus NPs (63–68 kDa) are indicated (black arrows). (**a**,**b**) Left panels: FLAG tag in red (secondary antibody: IRDye 680RD Donkey anti-mouse); middle panels: MTS-NP in green (secondary antibody: IRDye 800CW Donkey anti-rabbit); right panels: merged image. (**c**) Left panel: HA tag in red (secondary antibody: IRDye 680RD Donkey anti-mouse); middle panel: MTS-NP in green (secondary antibody: IRDye 800CW Donkey anti-rabbit); right panel: merged image. (**d**) Left panel: FLAG tag in red (secondary antibody: IRDye 680RD Donkey anti-mouse); middle panel: Hartmani-NP in green (secondary antibody: IRDye 800CW Donkey anti-rabbit); right panel: merged image. Immunodetection was performed using the Odyssey Infrared Imaging System (LICOR, Biosciences) providing also the molecular marker (Precision Plus Protein Dual Color Standards, Bio-Rad) used. Full-length blots are presented in Supplementary Fig. S3.

Following mitochondrial translocation, specific peptidases cleave the MTS of proteins destined to the mitochondrial matrix^33^. To investigate whether the putative MTSs of arenavirus NPs are cleaved, we studied boid brain (V/4Br) cells transfected with wtUGV1-NP-FLAG, mutUGV1-NP-FLAG, wtJUNV-NP-HA and mutJUNV-NP-HA as well as UHV1-NP-FLAG, LCMV-NP-HA and HISV1-NP-FLAG at three dpt by immunoblotting (Fig. 3a–d; see Supplementary Fig. S3a–d for uncropped blots). The same protein amount was loaded in the immunoblots for whole-cell lysates and mitochondrial preparations, and tubulin and mitofusin-2 (MFN2) served as cytosolic and mitochondrial markers, respectively (Ponceau S staining of the membranes, Supplementary Fig. S4a–d). To ensure an equal total protein amount, a much higher amount of cells was required for isolated mitochondria than for the whole-cell lysates. Therefore, MFN2 was enriched in the mitochondrial fractions and was barely detectable in the whole-cell lysates. Tubulin was abundant in the whole-cell lysates and could also be detected in the mitochondrial fractions, albeit at low level, due to the mild cytosolic contamination that is inevitable in the methodology used. Both wt and mutNPs produced a single main band with anti-FLAG or anti-HA antibodies in whole-cell lysates as well as in isolated mitochondria; based on the estimated molecular weight (approx. 63–68 kDa) this represents non-cleaved NP (Fig. 3a–d) which was also confirmed for HISV-1 NP by employing anti-Hartmani-NP antiserum (Fig. 3d).

To further investigate the fate of the NP’s N-terminal region, we produced an antiserum against the putative MTSs of rept- and mammarenavirus NPs. To demonstrate that the N terminus remains intact, i.e. the cleavage does not occur, we immunoblotted using the anti-MTS-NP antiserum. This reacted with bands of identical mobility as the anti-NP antiserum (Fig. 3a–c), thus confirming that the putative MTS remains non-cleaved. Furthermore, the immunoblotting revealed that both non-cleaved wt and mutNPs migrated in the mitochondrial fractions (Fig. 3a–d). Similar results regarding enrichment of NP localization in the mitochondrial fractions were obtained with reptarenavirus-infected I/1Ki and V/4Br cells (Supplementary Fig. S5a–d; Ponceau S stainings of the membranes, Supplementary Fig. S5e–h; see also Supplementary Fig. S6a–d for uncropped blots), using both the same methodology applied for transfected cells at one, two and four dpi (Supplementary Fig. S5a,b) and an alternative kit-based protocol for subcellular fractionation at one and two dpi (Supplementary Fig. S5c,d). Tubulin served as marker for the cytosolic fraction, and MFN2, mitochondrial ribosomal protein S35 (MRPS35), voltage-dependent anion-channel (VDAC), and mitochondrial outer membrane translocase 20 (TOM20) as mitochondrial markers. The results support the immunoblot results obtained from transfected cells regarding mitochondrial localization of a subpopulation of NP.

**Figure 4:**
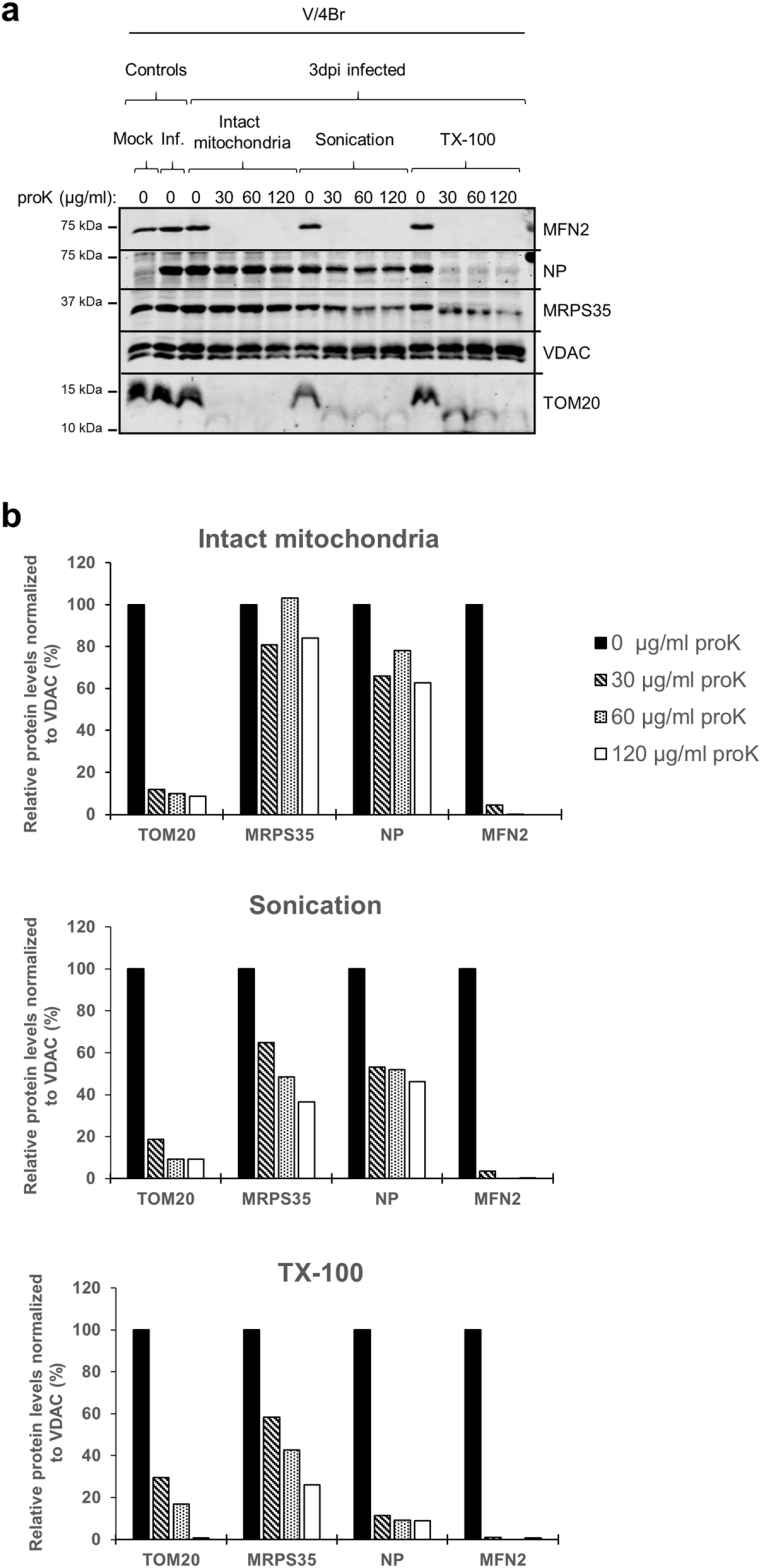
Submitochondrial analyses of reptarenavirus NP in infected boid cells. (**a**) Submitochondrial localization assay, protein localization as determined by protease accessibility. Mitochondria were isolated at three dpi from reptarenavirus-inoculated *Boa constrictor* V/4Br cells and either treated directly with proK at 30, 60 or 120 µg/ml, or subjected to sonication or TX-100 lysis first, and then treated with proK. For each condition, a sample without proK-treatment is provided as a control. An uninfected (Mock) and a reptarenavirus-infected (Inf.) mitochondrial sample at three dpi serve respectively as negative and positive controls for the anti-UHV-NP antibody used to detect the reptarenaviral NP. The samples (25 µg/lane of protein derived from the mitochondrial fraction) were separated through standard SDS-PAGE followed by immunoblotting to detect TOM20 and MFN2 (markers of the OMM), MRPS35 (marker of the mitochondrial matrix), VDAC (loading control) and reptarenaviral NP. Nitrocellulose membrane was cut into upper, middle and lower parts, that were subsequently incubated with antibodies against: upper part, rabbit affinity-purified anti-UHV-NP 1:500 (secondary antibody: IRDye 800CW Donkey anti-rabbit), followed by mouse anti-MFN2 1:200 (secondary antibody: IRDye 680RD Donkey anti-mouse); middle part, rabbit anti-MRPS35 1:1000 (secondary antibody: IRDye 800CW Donkey anti-rabbit), followed by mouse anti-VDAC 1:500 (secondary antibody: IRDye 680RD Donkey anti-mouse); lower part, rabbit anti-TOM20 1:1000 (secondary antibody: IRDye 800CW Donkey anti-rabbit). VDAC, embedded in the OMM membrane, is not affected by proK treatment and thus provides an internal reference for loading. Immunodetections were performed using the Odyssey Infrared Imaging System (LICOR Biosciences), showing also the molecular weight marker (Precision Plus Protein Dual Color Standards, Bio-Rad) used. (**b**) Quantification of the protein signals from the blot of Fig. 4a was performed by normalizing the signal intensity (determined through Image Studio Lite software, LICOR, Biosciences) of each band of TOM20, MRPS35, reptarenavirus NP and MFN2 to the corresponding one of VDAC, representing the internal loading control. For each condition of intact mitochondria (upper graph), sonication (middle graph) and TX-100 treatment (lower graph), the levels of each protein in proK-treated samples are presented relative to the level of the corresponding proK-untreated sample. Full-length blots are presented in Supplementary Fig. S7.

Given that the approaches to IB purification are, like those used to isolate mitochondria, based on centrifugation^20,22^, mitochondrial isolation conditions theoretically allow co-purification of NP-containing IBs. Therefore, we performed protease-based submitochondrial localization analyses^34^ (Fig. 4a,b; Ponceau S staining of the membrane in Supplementary Fig. S7a; see Supplementary Fig. S7b for uncropped blots) to provide further evidence that a fraction of NP indeed resides within mitochondria. We compared the effect of increasing proteinase K (proK) concentrations on intact, sonicated, and Triton X-100 (TX-100)-treated mitochondria, freshly isolated at three dpi from infected V/4Br cells and analyzed the samples by immunoblotting (Fig. S4a,b). While proteins localized at the cytosolic side of the outer mitochondrial membrane (OMM) are supposedly accessible for proK under all tested conditions, the proteins in the intermembrane space, on the inner mitochondrial membrane (IMM) and in the matrix are inaccessible to proK in intact mitochondria. Sonication and TX-100 treatment respectively served as mild and robust way of disrupting the mitochondrial membranes to render all mitochondrial proteins susceptible to proK degradation. The results show degradation of TOM20 and MFN2, proteins located in the OMM, under all conditions (Fig. 4a,b). TOM20 and MFN2 localization and topology are consistent with their degradation pattern: TOM20 is a small protein representing one of the receptors of the translocase of OMM (TOM) complex^31^, and MFN2 mediates mitochondrial fusion^35^; both proteins are widely exposed at the cytosolic side^31,35,36^. A lack of competing substrates in intact mitochondria could explain the slower degradation of TOM20 following sonication and TX-100 treatment. VDAC, also known as porin, is a β-barrel transmembrane channel that facilitates the exchange of metabolites and ions between the cytoplasm and the mitochondrial intermembrane space^37^. VDAC is firmly embedded in the OMM^37^; it therefore appeared inaccessible to proK under all test conditions (Fig. 4a,b) and served as internal loading reference. Unfortunately, we did not find a suitable marker protein for the intermembrane space of snake mitochondria. MRPS35 is a component of the small ribosomal subunit (mt-SSU) 28S of the mitochondrial ribosomes in the mitochondrial matrix and is known in humans to be localised on its surface^38^. MRPS35 remained intact upon proK treatment of intact mitochondria, whereas it was partially degraded following sonication and TX-100 treatment (Fig. 4a,b). ProK treatment of intact mitochondria resulted in degradation of a small proportion of reptarenavirus NP, as indicated by the quantification of NP protein (Fig. 4b) and the comparison to MRPS35 quantities, suggesting that some cytosolic NP co-purifies with the mitochondria. Sonication also resulted in slight NP degradation, while almost complete loss of NP was seen after TX-100 treatment (Fig. 4a,b). The degradation of MRPS35 after TX-100 treatment was slower than that of NP. This can be because MRPS35, as a component of the ribosome protein complex, is less accessible to degradation. Moreover, the mitochondrial ribosome also contains RNA components^38^ that might provide further protection from proteolysis. The results indicate similar degradation patterns for MRPS35 and NP, adding to the ultrastructural evidence (Fig. 1a–k; Supplementary Fig. S1a–d) that NP localizes in the mitochondrial matrix.

### NP mitochondrial localization is independent of a putative MTS also in mammalian cells

**Figure 5:**
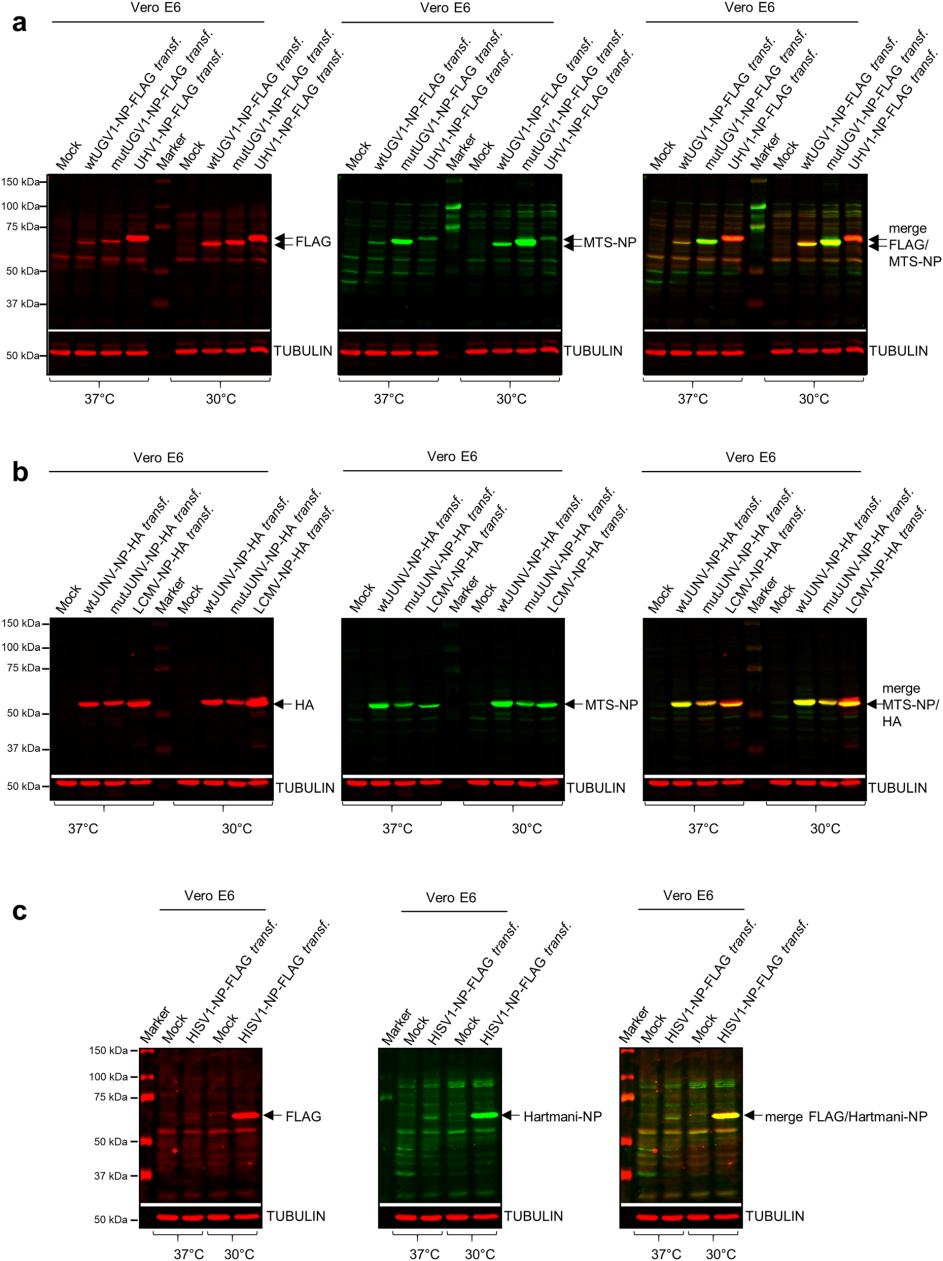
Immunoblotting studies on arenaviral NPs in transfected mammalian cells. (**a**–**c**) Immunoblotting analyses of whole-cell lysates obtained from monkey Vero E6 cells transfected with a construct expressing either wt or mutUGV1-NP-FLAG, or UHV1-NP-FLAG (**a**), wt or mutJUNV-NP-HA, or LCMV-NP-HA (**b**), HISV1-NP-FLAG (**c**), at three dpt after incubation at either 37 °C or 30 °C. Non-transfected (Mock) samples are provided as negative controls. 40 µg of protein per sample were loaded on standard SDS-PAGE gels, followed by immunoblotting. The nitrocellulose membranes were incubated sequentially with the following antibodies in the presented order: (1) mouse anti-FLAG tag 1:500 (**a**,**c**) or mouse anti-HA tag 1:500 (**b**); (2) rabbit anti-MTS-NP 1:200 (**a**,**b**) or rabbit anti-Hartmani-NP 1:500 (**c**); (3) mouse anti-tubulin 1:500 (**a–c**). Anti-tubulin specific signal at known molecular weight did not require membrane stripping. Tubulin (in red, secondary antibody: IRDye 680RD Donkey anti-mouse) was used as a reference for loading. Reptarenavirus and hartmanivirus (65–68 kDa), and mammarenavirus (63–65 kDa) NPs are indicated (black arrows). (**a**) Left panel: FLAG tag in red (secondary antibody: IRDye 680RD Donkey anti-mouse); middle panel: MTS-NP in green (secondary antibody: IRDye 800CW Donkey anti-rabbit); right panel: merged image. (**b**) Left panel: HA tag in red (secondary antibody: IRDye 680RD Donkey anti-mouse); middle panel: MTS-NP in green (secondary antibody: IRDye 800CW Donkey anti-rabbit); right panel: merged image. (**c**) Left panel: FLAG tag in red (secondary antibody: IRDye 680RD Donkey anti-mouse); middle panel: Hartmani-NP in green (secondary antibody: IRDye 800CW Donkey anti-rabbit); right panel: merged image. Immunodetection was performed using the Odyssey Infrared Imaging System (LICOR, Biosciences) providing also the molecular marker (Precision Plus Protein Dual Color Standards, Bio-Rad) used. Full-length blots are presented in Supplementary Fig. S8.

To rule out that reptilian cells are unable to mediate the processing of the putative MTS of NP, we studied mammalian cells transfected with the plasmids. The experiments were also intended to serve as controls for the mammarenavirus NPs (JUNV, wt and mutated, and LCMV). Therefore, we transfected African green monkey kidney (Vero E6) cells with the same constructs. Because we earlier demonstrated that reptarenaviruses replicate at 30 °C rather than at 37 °C^27^, we performed the experiments at both temperatures. Immunoblotting of whole-cell lysates at three dpt using anti-FLAG or anti-HA antibodies showed that all wt and mutNP constructs produced a protein of approximately 63–68 kDa in molecular weight at both temperatures (Fig. 5a–c; see Supplementary Fig. S8a–c for uncropped blots). We observed faster migration of wt vs mutNPs which is likely due to changes in the charge of the protein, since in the mutNPs the positively charged amino acids of the putative MTS were replaced by negatively charged amino acids. For HISV-1 NP we confirmed the result also by using the anti-Hartmani-NP antibody (Fig. 5c). Re-probing of the membranes with anti-MTS-NP antiserum showed that the putative MTSs remained uncleaved (Fig. 5a,b), as observed for the reptilian cells (Fig. 3a–c). Notably, the expression of both wt and mutUGV1 NPs as well as LCMV NP was stronger at 30 °C than at 37 °C (Fig. 5a,b). The phenomenon was most evident for HISV-1 NP which was barely detectable after incubation at 37 °C (Fig. 5c). To further demonstrate that the immunoblots on whole-cell lysates were sufficient to prove absence of MTS NPs processing in mammalian cells, we combined isolation of whole-cell lysates with mitochondria preparations of Vero E6 cells transfected with wt- and mutUGV1-NP-FLAG, or wt- and mutJUNV-NP-HA at three dpt. Transfections were performed at 37 °C to avoid perturbation of normal mammalian cell functions. Immunoblotting using anti-FLAG or anti-HA antibodies showed that the wt and mutNP constructs produced proteins of comparable molecular weight (63–68 kDa) in both whole-cell lysates and mitochondrial preparations, indicating NP mitochondrial localization also in mammalian cells and demonstrating that the putative MTS was not processed in isolated mitochondria (Supplementary Fig. S9a,b; Ponceau S staining of the membranes in Supplementary Fig. S9c,d; see Supplementary Fig. S9e,f for uncropped blots) and thereby confirming the previous observations (Fig. 5a–c). We observed very faint bands with anti-HA antibody at around 45 kDa in the mitochondrial preparations from transfections with both wt- and mutJUNV-NP-HA, but did not consider these as the result of putative MTS cleavage because: (1) most mitochondrial NP was still detected at the predicted molecular weight of the unprocessed protein, which would not be the case if MTS cleavage would be required for NP mitochondrial import; (2) the estimated 45 kDa size would require processing of about 150 amino acids, and MTSs are 20–70 amino acids long; (3) proteolytic cleavage during isolation by some proteases or "leaky scanning translation” can be possibilities.

### Mutations to the putative MTS alter the localization of NP

**Figure 6:**
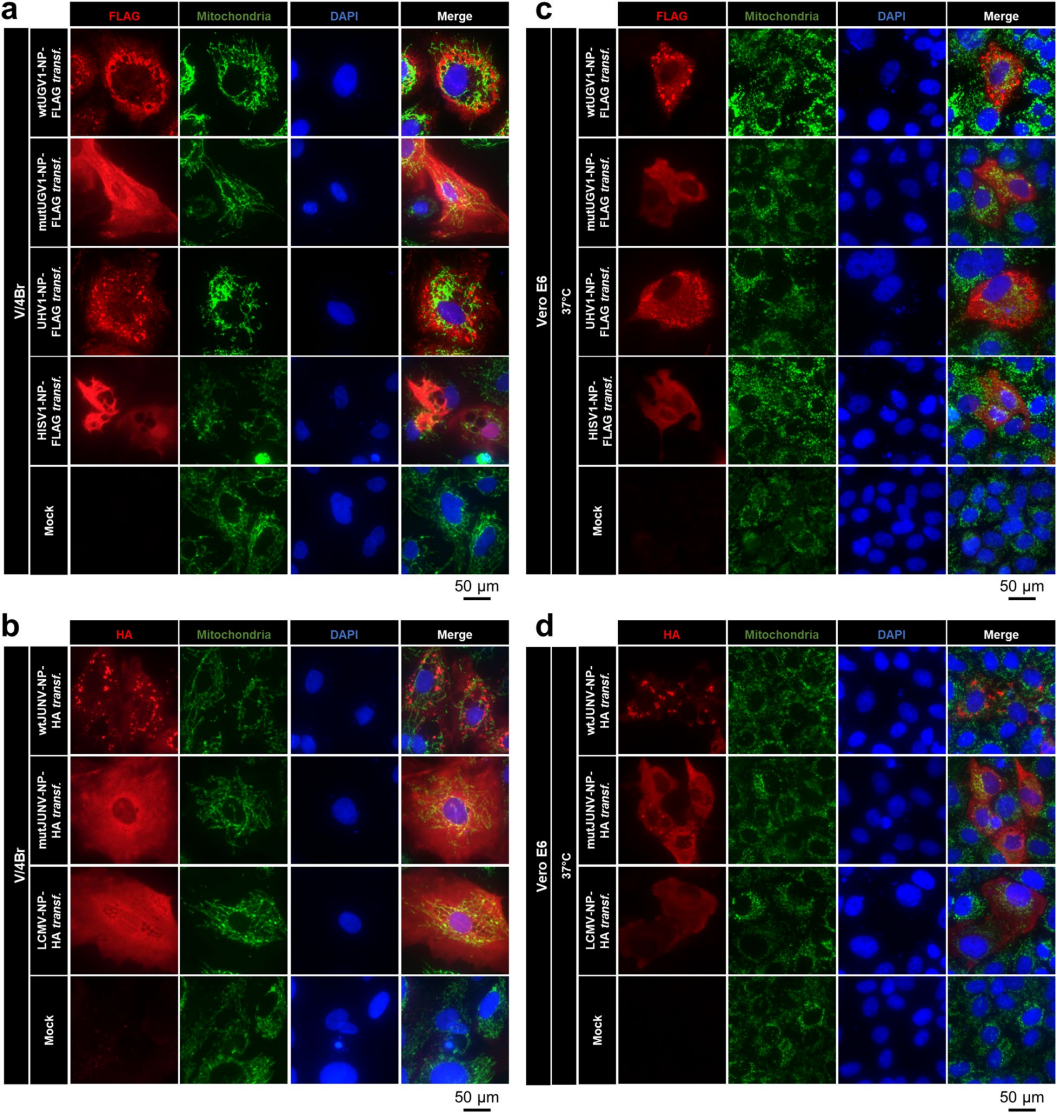
IF studies on arenaviral NPs in transfected boid and mammalian cells. Double IF images of *Boa constrictor* V/4Br cells incubated at 30 °C (**a**,**b**) or of monkey Vero E6 cells incubated at 37 °C (**c**,**d**), transfected with a construct expressing either wt or mutUGV1-NP-FLAG, UHV1-NP-FLAG, or HISV1-NP-FLAG (**a**,**c**), and wt or mutJUNV-NP-HA, or LCMV-NP-HA (**b**,**d**) at three dpt. Non-transfected (Mock) cells served as controls. (**a**,**c**) The panels from left: FLAG tag in red (secondary antibody: AlexaFluor 594 goat anti-rabbit), mitochondrial marker in green (secondary antibody: AlexaFluor 488 goat anti-mouse), nuclei in blue (DAPI), and a merged image. (**b**,**d**) The panels from left: HA tag in red (secondary antibody: AlexaFluor 594 goat anti-rabbit), mitochondrial marker in green (secondary antibody: AlexaFluor 488 goat anti-mouse), nuclei in blue (DAPI), and a merged image.

We further explored the mutations to the putative MTS of NPs with regards to their effect on the subcellular localization of NP and the potential to aggregate, which is a prerequisite of IBs formation and is commonly observed in reptarenavirus-infected and JUNV-infected cells^19,39,40^, as well as in NP-transfected cells analyzed by both TEM and immuno-EM (Fig. 1e–i). At three dpt, immunofluorescence (IF) analyses on *Boa constrictor* V/4Br cells transfected with wtUGV1-NP-FLAG, mutUGV1-NP-FLAG, wtJUNV-NP-HA and mutJUNV-NP-HA constructs showed for wtNPs either inclusions or aggregates giving a punctate/granular staining pattern with multifocal cytoplasmic accumulation within the cells, whereas the MTS-mutated NPs appeared disseminated throughout the cytoplasm of the transfected cells (Fig. 6a,b). We use the term “aggregates” to define irregularly distributed structures that are non-spherical and smaller than IBs, to differentiate them from the characteristic dense IBs. Transfection with UHV1-NP-FLAG consistently resulted in a staining pattern similar to the one of wtUGV1 and wtJUNV NPs (Fig. 6a), whereas LCMV NP was disseminated within the cells (Fig. 6b). Interestingly, HISV-1 NP was found disseminated within the cells or formed perinuclear tubular structures (Fig. 6a), confirming our earlier IF, TEM and immuno-EM findings in *Boa constrictor* I/1Ki cells^8^. To compare the expression pattern of arenaviral NPs in mammalian vs reptilian cells, we performed IF analyses on Vero E6 cells transfected with the same constructs at three dpt, after incubation at 37 °C (Fig. 6c,d) or 30 °C (Supplementary Fig. S10a,b). At 37 °C the results were comparable to those obtained in reptilian cells incubated at 30 °C (Fig. 6a,b). However, at 30 °C, the differences in the staining patterns between wtNPs and mutNPs were not apparent anymore; instead, a disseminated staining was observed for all NPs except HISV-1 NP (Supplementary Fig. S10a,b). For wtUGV1, wtJUNV and mutJUNV NP it was combined with some accumulation of inclusions/aggregates (white arrowheads, Supplementary Fig. S10a,b) that were less defined than those observed at 37 °C (Fig. 6c,d). The IF results for HISV-1 NP concurred with the immunoblot (Fig. 5c), a weak and disseminated staining pattern was seen in cells incubated at 37 °C (Fig. 6c) whereas cells kept at 30 °C presented more intense staining with tubular structures around the perinuclear area (Supplementary Fig. S10a) as previously described in reptilian cells^8^ (Fig. 6a).

For both V/4Br cells and Vero E6 cells at both 37 °C and 30 °C, IF analyses using the anti-MTS-NP antibody produced a disseminated staining pattern in cells transfected with the mutNP constructs, overlapping with the NP staining (Supplementary Fig. S11a–d). Curiously, cells expressing wtNPs, including LCMV NP displaying a disseminated pattern, showed no staining with anti-MTS-NP antibody (Supplementary Fig. S11a–d). Because with anti-MTS-NP only mutNPs were detected in IF, whereas both wt and mutNPs were recognized by this antibody via immunoblotting (Figs. 3a–c, 5a,b), we speculate that the tertiary structure of wtNPs might render the N terminus inaccessible to epitope recognition.

When we included the anti-mitochondria antibody into the IF analyses to locate the arenavirus NPs in relation to mitochondria, there was no clear evidence of co-localization, neither for wt nor mutNP variants in the cell lines tested (Fig. 6a–d, Supplementary Fig. S10a,b). Nevertheless, some wtNP inclusions/aggregates occasionally appeared to follow the mitochondrial network (Fig. 6a–d). This “anecdotal” co-localization is compatible with the ultrastructural observations which showed that only a proportion of mitochondria develop inclusions, and that mitochondria with NP often lose their structural integrity (Fig. 1a–k; Supplementary Fig. S1a–d), providing evidence of a potential loss of the mitochondrial marker used to detect the mitochondrial network. Furthermore, in Vero E6 cells after incubation at 30 °C (Supplementary Fig. S10a,b) the mitochondrial network appeared condensed. The suboptimal cultivation temperature could explain the slight differences observed between the distribution patterns of the analyzed arenavirus NPs in reptilian vs mammalian cells incubated at 30 °C (Fig. 6a,b; Supplementary Fig. S10a,b).

### Mitochondrial translocation of NP does not occur in vitro

**Figure 7:**
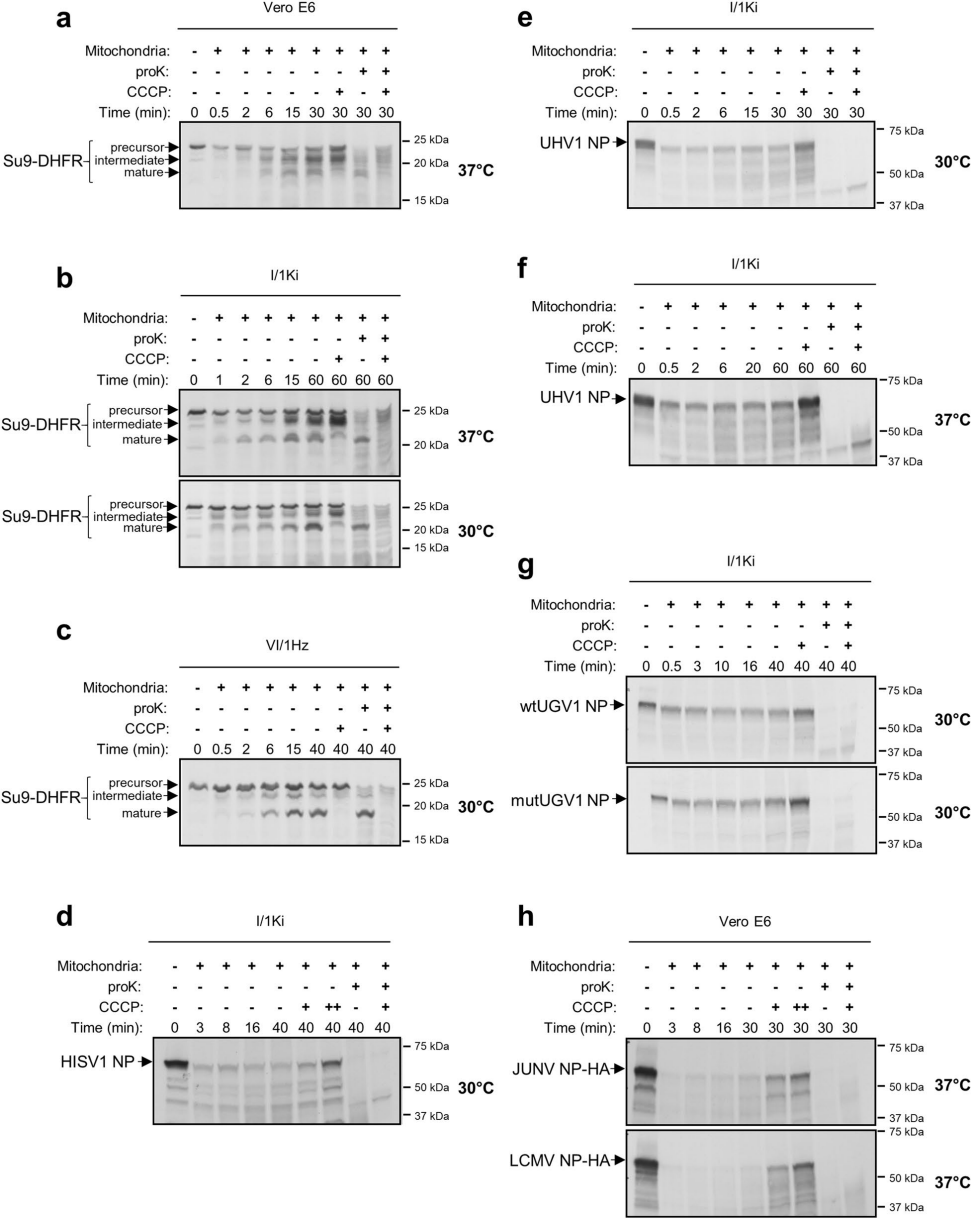
In vitro mitochondria import assay of arenaviral NPs. (**a**–**c**) The in vitro import into mitochondria was determined for a known chimeric mitochondrial protein, Su9-DHFR, used as positive control for the assay. The fusion protein was synthesized using [^35^S]-methionine in a rabbit reticulocyte lysate, from its sequence cloned in a pGEM4Z vector, flanking the SP6 RNA promoter. The hybrid protein was imported into freshly isolated mitochondria of monkey Vero E6 cells, at 37 °C (**a**), *Boa constrictor* kidney (I/1Ki) cells, at both 37 °C and 30 °C (**b**), and *Python regius* heart (VI/1Hz) cells, at 30 °C (**c**), as indicated by the presence at different time points of three distinct translocation forms: precursor, intermediate and mature forms (black arrows). (**d**–**h**) The in vitro translocation into freshly isolated *Boa constrictor* I/1Ki mitochondria was assessed for HISV-1 NP, at 30 °C (**d**), UHV-1 NP, at 30 °C (**e**) and 37 °C (**f**), wt and mutUGV-1 NPs, at 30 °C (**g**) and HA-tagged JUNV and LCMV NPs, at 37 °C (**h**). Radiolabelled NPs were in vitro synthesized using [^35^S]-methionine in a rabbit reticulocyte lysate, from their ORFs cloned into pGEM4Z (**d–g**) or pCR4Blunt-TOPO (**h**) vectors, flanking the SP6 (**d–g**) or T7 (**h**) promoter. Protein signals were determined through autoradiographic detection. CCCP: mitochondrial protein import blocker by inducing mitochondrial membrane potential dissipation. proK: leading to degradation of non-imported proteins. Full-length autoradiographies are presented in Supplementary Fig. S12.

To understand the mechanism behind the mitochondrial translocation of arenavirus NPs, we performed an in vitro mitochondrial import assay utilizing mitochondria freshly isolated from I/1Ki, python heart (VI/1Hz) and Vero E6 cells. First, we compared the ability of I/1Ki, VI/1Hz and Vero E6 cells to mediate the import of an in vitro translated chimeric mitochondrial fusion protein, Su9-DHFR (MTS of Subunit 9 of mitochondrial ATPase, Su9, of *Neurospora crassa* fused with the dihydrofolate reductase, DHFR, of *Mus musculus*^41,42^). The subsequent analysis identified the precursor, intermediate and mature mitochondria-imported forms of Su9-DHFR in the samples generated with mitochondria isolated from all tested cell lines (Fig. 7a–c; see Supplementary Fig. S12a–c for uncropped autoradiographies). Carbonyl cyanide m-chlorophenyl hydrazone (CCCP) treatment induces loss of mitochondrial membrane potential, thus significantly decreasing or abolishing MTS-mediated mitochondrial import^43^, and only the Su9-DHFR precursor and intermediate forms could be detected following the treatment (Fig. 7a–c). ProK treatment prior to electrophoresis led to loss of the Su9-DHFR precursor and intermediate forms in samples with intact (CCCP-untreated) mitochondria, while the mature form was protected from degradation as it was located within the mitochondria; all bands were lost following proK treatment of CCCP-treated samples (Fig. 7a–c). The Su9-DHFR translocation occurred at both 30 °C and 37 °C with similar efficacy (Fig. 7a–c). These results indicate that mitochondria isolated from cultured snake cells remain intact, and that the mitochondrial import system functions in a similar way in reptilian and mammalian cells.

Next we assessed the mitochondrial import of wtUGV-1 NP, mutUGV-1 NP, UHV-1, HISV-1, JUNV (wt) and LCMV NP and produced the proteins via in vitro coupled transcription/translation of their DNA sequences flanking either the SP6 or T7 promoter. The result indicates that arenavirus NPs cannot be translocated into mitochondria in vitro, since none of the proteins remained detectable following proK treatment (Fig. 7d–h; Supplementary Fig. S13a–d, see Supplementary Figs. S12d–h, S14a–d for uncropped autoradiographies).

## Discussion

Reptarenavirus NP is the main component of IBs pathognomonic to BIBD. Ultrastructural studies on reptarenavirus infected cells revealed IBs within mitochondria and immuno-EM confirmed the mitochondrial IBs to contain reptarenavirus NP. Strikingly, by studying the brain of a snake with BIBD by TEM and immuno-EM, we could demonstrate that mitochondrial translocation of reptarenavirus NP occurs also in vivo. These morphological findings prompted us to study the phenomenon and mechanisms of the potential mitochondrial transport of arenavirus NPs in more detail. Software prediction tools identified a putative MTS in the N terminus of rept- and mammarenavirus but not in hartmanivirus NPs. Analysis of mitochondria isolated from transfected cells suggested also that the NPs not only of reptarenaviruses, but also of hartmani- and mammarenaviruses, might enter the mitochondria, leading us to hypothesize that the feature is common among arenaviruses. Moreover, the absence of MTS cleavage and the fact that mutations to the putative MTS did not alter mitochondrial localization suggested that mitochondrial translocation would rather occur via an internal translocation signal (ITS). The ultrastructural examinations including immuno-EM allowed us to identify mitochondrial structures based on their characteristic electron density, texture and membrane structures. These considered the general morphological variability of mitochondria, which is influenced by, for example, their size, the level of sectioning and the biological state (fission/fusion), and therefore required a stringent comparison between mock infected controls and virus infected specimens. Interpretation of any reptarenaviral effects on the cells also took into consideration that the cultured cell populations comprise cells in variable growth phases, that only a proportion of mitochondria is affected in an infected cell at any given time, and that fission/fusion of mitochondria is a very rapid reaction (possibly occurring within a few minutes) which hampers the demonstration/interpretation of these effects. Ultimately, however, the ultrastructural findings provided proof that NPs localizes in and structurally affects mitochondria.

Our experiments showed that both wt and mutated forms of rept- and mammarenavirus NPs did not result in putative MTS cleavage. Although MTS-cleavage commonly occurs following MTS-mediated mitochondrial translocation^33^, the mitochondrial import can occur without MTS cleavage, e.g. the 10 aa MTS sequence of the human T-cell leukemia virus type 1 (HTLV-1) p13II protein remains intact during import^44^. In addition to the absence of MTS cleavage, we could not prevent mitochondrial localization by introducing mutations to the putative MTS, even though the mutations significantly lowered the software-predicted mitochondrial translocation potential. Not all proteins enter the mitochondria with the help of the classical N-terminal MTS; there might be additional internal cryptic elements within the NP that contribute to the mitochondrial localization. For instance, Hepatitis B virus (HBV) Pol protein localizes to both cytosol and mitochondria, and it can enter the mitochondria even after removal of its intrinsic MTS, suggesting the presence of multiple MTSs^45^. It is possible that the centrifugation-based methods applied herein for mitochondria isolation result in co-purification of IBs^20,22^. However, the submitochondrial localization studies based on protease accessibility support the immuno-EM findings that a subpopulation of NP remains protease treatment-resistant due to localization within mitochondria. Mutagenesis of the N-terminal putative MTS in UGV-1 and JUNV NP led to a diffuse distribution of the proteins. We propose this to indicate involvement of the NP’s N terminus to homo-oligomerization. This is supported by studies on Lassa virus (LASV) NP and LCMV NP, showing that for both the N-terminal region has been associated to homo-oligomerization^46,47^. Moreover, the lack of putative MTS cleavage in the mitochondrial fraction-localized NPs is compatible with IB-formation within mitochondria.

The folding and oligomerization status of NP could for instance affect the availability of the mitochondrial translocation signal (terminal or internal). Dual protein localization is a dynamic process responding to cellular conditions or aiming at rebalancing the distribution of protein subpopulations between cellular compartments. The subcellular localization of dually localized proteins depends on the accessibility of the protein’s targeting signal, determined by folding, proteolytic cleavage, binding to other proteins, and post-translational modifications^48^. The software predictions for arenavirus NP would fit its dual localization to cytosol and mitochondria. We thus speculate that the mitochondrial translocation signal of arenavirus NP would be available in the monomeric form of the protein. An example of viral proteins with multiple localization is given by the Human Herpesvirus-8 (Kaposi’s sarcoma-associated herpesvirus), which harbors two anti-apoptotic proteins: the K7 protein localizes to ER, nucleus and mitochondria, and the KS-Bcl2 protein to either mitochondria or nucleus. In addition, the folding state of the NP could induce its reverse translocation back to the cytoplasm during the import process, as described e.g. for the subcellular localization of the *Saccharomyces cerevisiase* fumarase^49^. Alternatively, RNA binding could affect the mitochondrial translocation of NPs, e.g. only NPs free from RNA would enter the mitochondria. In addition, co-translational mitochondrial import, in which only the newly translated protein originating from ribosomes close to mitochondria becomes translocated^50^, might be another factor contributing to mitochondrial import of only a subpopulation of NP. However, we could not reproduce the mitochondrial translocation of NP in isolated mitochondria in vitro, which we speculate to indicate the need for other cellular factors. Alternative explanations include the fact that the protein synthesis occurred prior to addition of isolated mitochondria in the in vitro assay, which would have disallowed co-translational import.

Our results suggest NP entry and subsequent IB formation within mitochondria, which appear to damage the mitochondria. The NP's mitochondrial entry mechanism remains unknown, but we think that passive diffusion through damaged OMMs would be unlikely. In addition, TEM and immuno-EM (Fig. 1a–k, Supplementary Fig. S1a–d) showed that the morphological alterations of the mitochondria, due to IBs/NP, affect mostly the matrix and cristae of the IMM. Furthermore, NP is a 68 kDa protein, and IBs are substantially larger; for their passive migration to the mitochondrial matrix, overlapping permeability/breakage of both OMMs and IMMs would be required. Moreover, the mitochondrial membranes have a very different composition and, therefore, permeability^51^. While the OMM is permeable for up to 10 kDa solutes (ions, molecules and small proteins) thanks to the porin (VDAC) pores^37,51^, the IMM does not allow passive diffusion and requires regulated transporter mechanisms for the passage of proteins^51^.

The mitochondria play an essential role in the cell’s antiviral response. Their OMM houses the mitochondrial antiviral-signaling protein (MAVS) which mediates both the type I IFN response and pro-inflammatory pathways^52,53^. In addition, MAVS participates in the regulation of apoptosis^54,55^. Mitochondria are also involved in adaptive immunity via generation of reactive oxygen species (ROS), contributing to T cell activation, and via mitochondrial metabolism, regulating CD4+ /CD8+ (memory vs effector) T cell differentiation^4^. Mitochondrial targeting of NPs could contribute to the dampening of the innate immune response, thus adding to the NP’s function in preventing IFN signaling up- or downstream of MAVS^9–16^. The known examples of viruses targeting mitochondria as immune evasion strategy include MAVS signalosome degradation by severe acute respiratory syndrome coronavirus open-reading frame 9b protein^56^, and immune response suppression and activation of the nucleotide-binding domain (NOD)-like receptor protein 3 (NLRP3) inflammasome by the PB1-F2 protein of influenza A by altering the mitochondrial membrane potential^57^. We propose that future studies should address the role of (rept)arenavirus NP’s mitochondrial targeting in: (1) controlling mitochondrial immune response; (2) induction of mitophagy to avoid apoptosis; and (3) induction of mitochondrial biogenesis to control the metabolic and redox state of the cell.

## Methods

### Cell lines and viruses

The study made use of the African green monkey kidney Vero E6 (American Type Culture Collection [ATCC]) cell line, and permanent tissue cell cultures derived from *Boa constrictor* brain (V/4Br) and kidney (I/1Ki)^19^, and from *Python regius* heart (VI/1Hz). The cells were maintained in Minimum Essential media (MEM, Gibco) containing 10% fetal bovine serum (FBS, Biochrom), 10% tryptose phosphate broth (TPB, Difco), 6 mM Hepes (Biochrom), 2 mM l-alanyl-l-glutamine (Biochrom) and 50 µg/ml Gentamicin (Gibco). The snake cells were maintained at 30 °C and the mammalian cells at 30 °C or 37 °C, with 5% CO_2_.

For infections, the single UGV-1 isolate was used. The virus mix was prepared by inoculating I/1Ki cells at a multiplicity of infection (MOI) of 0.1 to 0.01, and by pooling the supernatants collected at three, six, nine and twelve dpi. The mix was stored in aliquots at − 80 °C, an MOI of 1–10 was used in the infection experiments.

### Plasmids and molecular cloning

The open-reading frames (ORFs) for wt UGV-1 NP (Gene ID: 37629387), UHV-1 NP (GeneID: 18821736) and HISV-1 NP (Gene ID: 41324517) were cloned into the pCAGGS-FLAG vector in frame with the C-terminal FLAG tag; the wt JUNV NP (Gene ID: 2545643) and wt LCMV NP (Gene ID: 956592) in pCAGGS-HA vector, in frame with the C-terminal HA tag^14^. wtJUNV NP, wtLCMV NP and the pCAGGS-FLAG and pCAGGS-HA vectors have been described earlier^14,58^. The N-terminal mutations to the putative MTS of UGV-1 NP (R6E, K15E, K16E, K17E, K20E) and JUNV NP (K5E, R11E, R17E, R18E) were generated using synthetic genes from Invitrogen which were subcloned into either pCAGGS-FLAG or pCAGGS-HA vectors in frame with the tag.

The NP ORFs of UGV-1 (wt and MTS-mutated), UHV-1, HISV-1, JUNV (wt) and LCMV were also subcloned into the pCR4Blunt-TOPO vector using the Zero Blunt TOPO PCR Cloning Kit for Sequencing, with One Shot TOP10 Chemically Competent *E. coli* (Thermo Fisher Scientific) following the manufacturer’s instructions. Individual clones were sent for Sanger sequencing at Microsynth Ag, and the plasmids containing the insert in coding orientation under the T7 RNA promoter were used for in vitro transcription/translation for the expression of radiolabelled NPs to assess mitochondrial import. The inserts were also subcloned into the pGEM4Z vector in coding orientation under the SP6 promoter (UGV-1, UHV-1, HISV-1, JUNV and LCMV NP), or under the T7 promoter for HISV-1 NP. The molecular cloning followed standard procedures, and the primers used are listed in Supplementary Table S2.

### Transfections

The transfections were performed as described^59^. Lipofectamine 2000 (Invitrogen) was used for snake cells, and FuGENE HD (Promega) for mammalian cells. Fresh conditioned medium was provided to the cells at 16–20 h post-transfection (hpt), and the analyses (immunoblot, IF, TEM and immuno-EM) were performed on cells collected at three dpt.

### Protein extraction from cultured cells

Trypsinized cells were washed twice with ice-cold PBS, pelleted by centrifugation (800×*g*, 7 min, 4 °C) and resuspended in ice-cold radioimmunoprecipitation assay (RIPA) buffer (50 mM tris, 150 mM NaCl, 1% TX-100, 0.5% Sodium deoxycholate, 0.1% sodium dodecyl sulphate [SDS], pH 8.0) supplemented with complete protease inhibitor cocktail (Roche). Following 30 min incubation on ice and 10 min sonication, the lysates were clarified by centrifugation (13,000×*g*, 10 min, 4 °C). The protein concentration was measured using the Pierce BCA Protein Assay Kit (Thermo Fisher Scientific), the lysates were stored at − 80 °C.

### Isolation of mitochondria from cultured cells

For isolation of mitochondria, confluent layers of cultured cells (surface area ≥ 150 cm^2^) were trypsinized, washed twice with ice-cold PBS, and pelleted by centrifugation (800×*g*, 7 min, 4 °C). The cell pellets were resuspended in 4 ml of ice-cold Mitochondria Isolation Buffer (MIB: 20 mM Hepes, 220 mM mannitol, 70 mM sucrose, 1 mM ethylenediaminetetraacetic acid [EDTA], pH 7.6) with 2 mg/ml fatty acid-free bovine serum albumin (BSA, Sigma), supplemented with EDTA-free complete protease inhibitor cocktail (Roche) and kept on ice for 15–20 min. The cell suspensions were transferred to 5 ml Dounce homogenizers and manually homogenized. The homogenates were centrifuged (800×*g*, 5 min, 4 °C) and the pellets subjected to another round of homogenization and centrifugation. The supernatants were transferred into new tubes and the mitochondria pelleted by centrifugation (10,000×*g*, 10 min, 4 °C). The pelleted mitochondria were washed with 4 ml of ice-cold MIB, re-pelleted by centrifugation (10,000×*g*, 10 min, 4 °C), and re-suspended in 80–600 µl of MIB. Mitochondrial preparations were stored at − 80 °C.

### Sodium dodecyl sulphate–polyacrylamide gel electrophoresis (SDS-PAGE) and immunoblot

The proteins from cell lysates and the isolated mitochondria were analyzed by separating 10–40 µg of protein (concentrations determined using Pierce BCA Protein Assay kit, Thermo Fisher Scientific) by SDS-PAGE. The samples were diluted in Laemmli Sample Buffer (LSB, final concentration: 0.3% SDS, 60 mM tris–HCl pH 6.8, 10% glycerol, 0.62% β-mercaptoethanol, 1% bromophenol blue) and denatured (10 min at 70 °C), then loaded on 7.5% or 12% SDS-PAGE gels. After SDS-PAGE under standard conditions, the proteins were wet transferred (either 400 mA for 2 h or 160 mA overnight) onto nitrocellulose membrane (0.45 µm, Amersham Protran) in transfer buffer (25 mM tris, 200 mM glycine, 20% methanol). The membranes were blocked in Tris-buffered saline (TBS)-T (50 mM tris, 150 mM NaCl, 0.05% Tween 20, pH 7.4) with 5% (w/v) BSA. After primary (overnight at 4 °C or 2–3 h at room temperature (RT)) and secondary (2 h at RT) antibody incubations and appropriate washes (3–5 times for 5 min with TBS-T after antibody incubations, and twice with TBS before detection), the results were recorded using the Odyssey Infrared Imaging System (LICOR Biosciences). If required, a stripping step with NewBlot Nitro Stripping Buffer 5X (LICOR, Biosciences) was perfomed (10 min at RT) between reactions with different antibodies.

For the mitochondrial import assay, arenavirus NP samples were separated on pre-cast 7.5% SDS-PAGE gels (Bio-Rad). Afterwards, gels were stained with the Coomassie stain (PhastGel Blue R, Sigma) to visualize the proteins. Gels were then fixed (20% ethanol and 10% acetic acid), treated with Amplify (Amersham) for 30 min and dried. Lysates obtained from import experiments performed on Su9-DHFR control were separated on 14% SDS-PAGE gels and transferred to nitrocellulose as described above. The [^35^S]-methionine-labelled proteins on either dried gels or nitrocellulose membranes were visualized by autoradiographic detection using Amersham Hyperfilm MP (GE Healtcare).

### Antibodies

The antibodies used are listed in Supplementary Table S3. The polyclonal rabbit antiserum against the putative MTSs of arenavirus NPs (named anti-MTS-NP) was raised against a synthetic multiepitope protein with the following sequence: MAALQRAAVNQLALKKKLNKMLSPFQRELNNQIFGGGGGMAALQEAAVNQLALEEELNEMLSPFQEELNNQIFGGGGGMSLSKEVKSFQWTQALRRELQGGGGGMSLSEEVESFQWTQALEEELQGGGGGMAAFQKAAVNQLALKKKLNKMLAPYQRELNNQIFGGGGGMAAFQEAAVNQLALEEELNEMLAPYQEELNNQIFGGGGGMAHSKEVPSFRWTQSLRRGLSGGGGGMAHSEEVPSFEWTQSLEEGLSHHHHHH. The polyclonal rabbit antiserum against hartmanivirus NPs (named anti-Hartmani-NP) was raised against a synthetic protein bearing amino acid stretches of HISV-1 NP with highest similarity to the NPs of other hartmaniviruses, sequence: EVLTNQLQVDYLFILIFCAKKQNMDLEALLELSGRCKLIFNKLPFTQKVLTQLSKSAKIESSIEDLVIFTQTGYLDEKYLRKQGSGKLAGFMAKQHGMTKECKHAAKGGGGGGYSILREIENNLVLHDSPFRLNRQRFQSAVSALTGCVSDRMVSSGGGGGCKHKDGITVNTSEGSTTTYELLLHSILTTPTINAKIKNRTNVRRNGLNTVRFIGGGHHHHHH. Synthetic genes (in pET20b(+) vector) encoding the proteins were ordered from GeneUniversal, and the proteins produced in *E.coli* and purified as described^27,60^. Immunizations and sera collections were performed by BioGenes GmbH as described^27,60,61^.

### IF staining

For IF staining cells were seeded onto 13 mm coverslips (Thermo Fischer Scientific) in 24-well plates. The reptarenavirus NP-transfected cells were PBS washed twice (at three dpt) and fixed with 4% PFA (in PBS) for 10–30 min at RT. After a PBS wash, cells were permeabilized and blocked (0.25% TX-100 and 0.5% (w/v) BSA in PBS) for 5–10 min at RT, and washed twice with PBS. The cells were incubated with the primary antibodies (overnight at 4 °C or 1 h at RT), washed 3–5 times with PBS, incubated 1 h at RT with the secondary antibodies, washed 3–5 times with PBS, incubated 15 min at RT with DAPI (Novus Biologicals, 1 µg/µl, diluted 1:10,000 in PBS), and washed twice with milli-Q water prior to mounting with FluoreGuard mounting medium (Scytek Laboratories). All primary and secondary antibodies were diluted in Dako REAL antibody diluent (Agilent technologies). Images were captured and analyzed using a Nikon Eclipse TI microscope with NIS-Elements Microscope Imaging Software (Nikon).

### TEM and immuno-EM

TEM and immuno-EM studies were performed on cells grown in chamber slides (ibidi) as described^8^ and brain samples collected from a euthanized *B. constrictor* with BIBD immediately after the animal’s death. Briefly, pelleted cells/tissue specimens were fixed in 1.5%/2.5% glutaraldehyde, buffered in 0.2 M cacodylic acid buffer, pH 7.3, for 12 h at 5 °C and routinely embedded in epoxy resin. Toluidin blue stained semithin sections (1.5 μm) and, subsequently, ultrathin (100 nm) sections were prepared and the latter contrasted with lead citrate and uranyl acetate and examined with a Philips CM10 transmission electron microscope at 80 kV.

For immuno-EM, ultrathin sections were incubated for 30 min at RT in PBS with 1% BSA, followed by overnight incubation with the primary antibody (Supplementary Table S3, diluted in PBS with 1% BSA) at 4 °C. After washing with PBS, sections were incubated with the secondary antibody (Supplementary Table S3, diluted in PBS with 1% BSA) for 2 h at RT, contrasted and examined as described above.

### Submitochondrial localization assay

The submitochondrial localization assay used in the study is based on protease accessibility and was performed as described^34,62^, with some modifications. Freshly isolated mitochondria from a confluent cell layer (≥ 750 cm^2^) were resuspended in MIT buffer (320 mM sucrose, 10 mM tris, 1 mM EDTA, pH 7.4). The protein concentration was assessed using the Pierce BCA Protein Assay kit (Thermo Fisher Scientific). The mitochondria were pelleted by centrifugation (14,000×*g*, 10 min, 4 °C), resuspended in MIT buffer to yield 10 µg/µl, and divided into 4 different fractions of 100 µl each: A, B1 and B2, and C. Subsequently, pelleted mitochondria (14,000×*g*, 10 min, 4 °C) of each fraction were resuspended in 400 µl MIT for fraction A, 400 µl sonication solution (500 mM NaCl and 10 mM tris, pH 7.4) for fraction B1, 400 µl sonication solution and 3.8 µg/µl of proK (Recombinant PCR Grade [Roche]) for fraction B2, and 400 µl MIT-T (MIT + 0.5% TX-100) for fraction C. The fractions B1 and B2 were sonicated on ice (three times for 30 s with 40% duty cycle in a Bandelin sonopuls sonicator) and fraction C was mixed by pipetting the solution up and down for 50 times. Fraction A represents intact mitochondria, fractions B1 and B2 vesicles obtained by sonicating mitochondria, and fraction C lysed mitochondria.

Fractions A and C were divided into four equal volume samples of 50–100 µl each. From fraction B1, a volume of 50–100 µl was taken and fraction B2 was divided into three equal volume samples of 50–100 µl each. MIT buffer (1/20 volume) without or with 30 µg/ml, 60 µg/ml or 120 µg/ml of proK was added to the samples, and after 20 min incubation on ice phenylmethylsulfonyl fluoride (PMSF, Sigma) was added to a final concentration of 2 mM, followed by 10 min incubation on ice. Then, 1% sodium deoxycholate was added to each sample to a final concentration of 0.05%, followed by 20 min incubation on ice. The proteins were precipitated by adding (1/6 volume) 100% (w/v) trichloroacetic acid (TCA), followed by 30 min incubation on ice and centrifugation (14,000×*g*, 30 min, 4 °C). The pelleted proteins were washed with 1 ml of ice-cold acetone, pelleted by centrifugation (14,000×*g*, 30 min, 4 °C), dried for 5 min at 37 °C, and resuspended in 2 × LSB to reach final concentration of 2.5 µg/µl. The assay was completed by analysing the samples via SDS-PAGE and immunoblot procedures, as previously described.

### Import of radiolabelled proteins into isolated mitochondria

The hybrid mitochondrial protein Su9-DHFR, comprising the fusion of MTS of Subunit 9 of mitochondrial ATPase (Su9) of *N. crassa* with the DHFR of *M. musculus*, served as positive control for the protein import into mitochondria. Su9-DHFR was expressed under the SP6 RNA promoter in the pGEM4Z vector^41,42^.

The mitochondrial protein import assay was carried out as described^43^, with some modifications. Full-length (tagged or untagged) NPs and Su9-DHFR were in vitro translated using rabbit reticulocyte lysate with either the TnT SP6 quick coupled Transcription/Translation System (Promega) for sequences flanking the SP6 promoter, or the TnT T7 quick coupled Transcription/Translation System (Promega) for sequences flanking the T7 promoter, following the manufacturer’s instructions, in the presence of 20 µCi [^35^S]-methionine (Hartmann Analytic). The mitochondria were freshly isolated for the experiments from I/1Ki, VI/1Hz or Vero E6 cells. Protein concentrations were determined by the Pierce BCA Protein Assay kit (Thermo Fisher Scientific). The mitochondria were divided into 75 µg preparations, each one for a specific incubation time point ± proK (Recombinant PCR Grade [Roche]) and/or CCCP addition, pelleted (10,000×*g*, 10 min, 4 °C), resuspendend in 100 µl import buffer (250 mM sucrose, 5 mM magnesium acetate, 80 mM potassium acetate, 20 mM Hepes–KOH, pH 7.4) supplemented with freshly added 10 mM sodium succinate, 5 mM adenosine triphosphate (ATP), and 1 mM dithiothreitol (DTT), and 5 µl of in vitro translation mix were added. CCCP used at 1 mM or 2 mM concentration served as import blocker. The import reactions were performed at 30 °C or 37 °C under continuous rotation; at the end of the incubation proK was added (final concentration 50 µg/ml) to degrade the non-imported proteins. After 15 min incubation on ice, proK was inactivated by adding PMSF at a final concentration of 2 mM, followed by 10 min incubation on ice. Next, the mitochondria were centrifuged (12,000×*g*, 5 min, 4 °C), washed with 200 µl SET buffer (250 mM sucrose, 10 mM tris–HCl pH 7.6, 1 mM EDTA, 0.1 mM PMSF), centrifuged (12,000×*g*, 5 min, 4 °C), and resuspended in 20 µl of 1 × LSB. Samples were loaded on SDS-PAGE gels and analyzed as described above.

### Ethical approval

The snake included in this study was privately owned and was submitted to the Institute of Veterinary Pathology, Vetsuisse Faculty, University of Zurich by its owner for euthanasia and subsequent diagnostic post mortem examination, due to the clinical suspicion of BIBD. The animal was euthanized according to the ASPA, Animals (Scientific Procedures) Act 1986, schedule 1 (appropriate methods of humane killing, http://www.legislation.gov.uk/ukpga/1986/14/schedule/1) procedure, and necropsied with full owner’s informed consent also to the use of the tissue samples for this study. For anesthesia 10–20 mg/kg Ketamin (0.1–0.2 ml/kg) and 0.1–0.2 mg/kg Medetomidin (0.1–0.2 ml/kg) were injected intramuscularly in the cranial third of the body. Once deep anaesthesia was achieved, after appr. 45 min, the animal was decapitated. For such diagnostic euthanasia and post mortem examination, no ethical permission is required, as both are routine veterinary procedures. Also, the terms of service to which owners agree when submitting an animal for a diagnostic post mortem examination include the permission to make use of material from the examination for both teaching and research.

## Supplementary Information

Supplementary Information.

## Data Availability

The datasets generated during and/or analyzed during the current study are available from the corresponding author on reasonable request.
