# Supplementary material for: A subpopulation of arenavirus nucleoprotein localizes to mitochondria
Source: Sci Rep. 2021 Oct 26;11:21048. doi: 10.1038/s41598-021-99887-5 (PMC8548533; doi:10.1038/s41598-021-99887-5)
Supplement: Supplementary file 1 — Supplementary Information. [file 41598_2021_99887_MOESM1_ESM.pdf]

Supplementary Information

A subpopulation of arenavirus nucleoprotein localizes to mitochondria

Francesca Baggio[1,2,*], Udo Hetzel[1,3], Lisbeth Nufer[1], Anja Kipar[1,3¶], Jussi Hepojoki[1,4¶]

[1]Institute of Veterinary Pathology, Vetsuisse Faculty, University of Zurich, 8057 Zurich, Switzerland

[2]Center for Clinical Studies, Vetsuisse Faculty, University of Zurich, 8057 Zurich, Switzerland

[3]Department of Veterinary Biosciences, Faculty of Veterinary Medicine, University of Helsinki, 00014 Helsinki, Finland

[4]Department of Virology, Medicum, Faculty of Medicine, University of Helsinki, 00290 Helsinki, Finland

[*]Corresponding author

E-mail: francesca.baggio@uzh.ch

[¶] These authors contributed equally to this work.

Supplementary Methods

Subcellular fractionation from cultured cells

Subcellular fractionation was done using the Cell Fractionation kit-Standard (ab109719, Abcam) following the manufacturer's instructions. For the experiment, the cells were seeded on 10 cm diameter cell culture dishes ($2.5*10^6$ cells/dish) and collected at a concentration of $6.6*10^6$ cells/ml. Reptarenavirus inoculation (MOI of 5-10) occurred immediately after the cells were seeded; non-inoculated cells served as controls. The cells were harvested for isolation at either one or two dpi. Samples of intact cells (Total fraction, T) were collected before mixing the cell suspension with detergent solutions used for cell fractionation. Before analyses, the cytosolic (C) and mitochondrial (M) fractions were 10-fold concentrated using Amicon Ultra-0.5 Centrifugal Filter Units (Merck Millipore) following the manufacturer's instructions.

Bioinformatics

MitoProt II (http://ihg.gsf.de/ihg/mitoprot.html) and TargetP 1.1 (http://www.cbs.dtu.dk/services/TargetP-1.1) served for determining the probabilities for mitochondrial localization and the predictions for MTS cleavage sites for arenavirus NPs.

Supplementary Tables

Supplementary Table S1: *In silico* predictions of mitochondrial localization for different arenaviral NPs

<table>
<tr><th rowspan="4">Arenaviridae genera</th><th rowspan="4">Virus
[NP GenBank Accession n.]</th><th rowspan="4">Construct (for transfections)</th><th colspan="8">Bioinformatic predictions of mitochondrial localization for arenaviral nucleoproteins (NPs)</th></tr>
<tr><th colspan="4">Target P 1.1</th><th colspan="4">Mitoprot II</th></tr>
<tr><th rowspan="2">% mt</th><th colspan="2">N-terminal MTS</th><th rowspan="2">RC</th><th rowspan="2">% mt</th><th colspan="3">Cleavage site</th></tr>
<tr><th>Length (aa)</th><th>MTS Mw (kDa)</th><th>Position (aa)</th><th colspan="2">MTS Mw (kDa)</th></tr>
<tr><td rowspan="10">Reptarenavirus</td><td rowspan="3">University of Giessen virus 1 (UGV-1) [AKN10674.1]</td><td>wtUGV1-NP</td><td>59.5</td><td>7</td><td>0.8</td><td>5</td><td>97.6</td><td>n.p.</td><td colspan="2">-</td></tr>
<tr><td>wtUGV1-NP-FLAG</td><td>59.5</td><td>7</td><td>0.8</td><td>5</td><td>97.5</td><td>n.p.</td><td colspan="2">-</td></tr>
<tr><td>mutUGV1-NP-FLAG</td><td>6.9</td><td>-</td><td>-</td><td>2</td><td>2.3</td><td>n.p.</td><td colspan="2">-</td></tr>
<tr><td rowspan="2">University of Helsinki virus 1 (UHV-1) [AGS94416.1]</td><td>UHV1-NP</td><td>46.2</td><td>-</td><td>-</td><td>5</td><td>93.1</td><td>n.p.</td><td colspan="2">-</td></tr>
<tr><td>UHV1-NP-FLAG</td><td>46.2</td><td>-</td><td>-</td><td>5</td><td>92.9</td><td>n.p.</td><td colspan="2">-</td></tr>
<tr><td>Aurora borealis virus 1 (ABV-1) [AKN10670.1]</td><td>-</td><td>59.4</td><td>47</td><td>5.4</td><td>5</td><td>97.1</td><td>n.p.</td><td colspan="2">-</td></tr>
<tr><td>Golden Gate virus 1 (GGV-1) [YP_006590091.1]</td><td>-</td><td>33.5</td><td>-</td><td>-</td><td>4</td><td>94.1</td><td>n.p.</td><td colspan="2">-</td></tr>
<tr><td>Tavallinen suomalainen mies virus 1 (TSMV-1) [APX61209.1]</td><td>-</td><td>53.7</td><td>7</td><td>0.8</td><td>5</td><td>89.9</td><td>n.p.</td><td colspan="2">-</td></tr>
<tr><td>Rotterdam reptarenavirus (ROUT) [AGH06040.1]</td><td>-</td><td>60.6</td><td>7</td><td>0.8</td><td>4</td><td>91.6</td><td>30</td><td colspan="2">3.2</td></tr>
<tr><td>California reptarenavirus (CASV) [AFP93552.1]</td><td>-</td><td>39.9</td><td>-</td><td>-</td><td>4</td><td>84.8</td><td>n.p.</td><td colspan="2">-</td></tr>
<tr><td rowspan="2">Hartmanivirus</td><td rowspan="2">Haartman Institute virus 1 (HISV-1) [AKN10684.1]</td><td>HISV1-NP</td><td>6.6</td><td>-</td><td>-</td><td>1</td><td>6.0</td><td>n.p.</td><td colspan="2">-</td></tr>
<tr><td>HISV1-NP-FLAG</td><td>6.6</td><td>-</td><td>-</td><td>1</td><td>5.8</td><td>n.p.</td><td colspan="2">-</td></tr>
</table>

	**Haartman Institute virus 2 (HISV-2) [AZI72594.1]**	-	7.5	-	-	1	8.1	n.p.	-
	**Old schoolhouse virus 1 (OScV-1) [AZI72576.1]**	-	5.7	-	-	1	5.4	n.p.	-
	**Old schoolhouse virus 2 (OScV-2) [AZI72582.1]**	-	5.7	-	-	1	5.4	n.p.	-
	**Veterinary Pathology Zurich virus 1 (VPZV-1) [AZI72592.1]**	-	6.9	-	-	1	7.9	n.p.	-
	**Veterinary Pathology Zurich virus 2 (VPZV-2) [AZI72596.1]**	-	6.8	-	-	1	6.2	n.p.	-
	**Dante Muikkunen virus 1 (DaMV-1) [AZI72578.1]**	-	7.5	-	-	1	14.3	n.p.	-
Antennavirus	**Wenling frogfish arenavirus 1 [AVM87642.1]**	-	7.9	-	-	2	2.7	n.p.	-
Mammarenavirus	**Junin virus (JUNV, or Argentianian virus) [AAU34181.1]**	**wtJUNV-NP**	69.5	61	7.0	4	83.4	28	3.2
		**wtJUNV-NP-HA**	69.5	61	7.0	4	83.5	28	3.2
		**mutJUNV-NP-HA**	5.4	-	-	1	2.3	n.p.	-
	**Lymphocytic choriomeningitis virus (LCMV) [AHZ55914.1]**	**LCMV-NP**	41.6	-	-	5	74.8	n.p.	-
		**LCMV-NP-HA**	41.6	-	-	5	74.6	n.p.	-
	**Machupo virus (MACV) [AIG51559.1]**	-	67.2	20	2.4	4	54.2	28	3.2
	**Lassa virus (LASV) [ADY11071.1]**	-	32.3	-	-	4	79.9	n.p.	-
	**Guaranito virus (GTOV) [AAT72112.1]**	-	42.3	-	-	5	19.8	n.p.	-
	**Pichinde virus (PICV, or Cali mammarenavirus) [AAC32282.1]**	-	78.3	25	3.0	3	65.6	26	3.0
	**Tacaribe virus (TCRV) [AHW46356.1]**	-	54.8	61	7.0	5	69.6	n.p.	-

For bioinformatic predictions of mitochondrial localization of arenaviral NPs belonging to the

four different genera (*Reptarenavirus*, *Hartmanivirus*, *Antennavirus* and *Mammarenavirus*), the bioinformatic programs TargetP 1.1 (http://www.cbs.dtu.dk/services/TargetP-1.1) and MitoProt II (http://ihg.gsf.de/ihg/mitoprot.html) were used. TargetP 1.1 reported data consist in: percentage of mitochondrial localization (% mt), length (amino acids, aa) and molecular weight (Mw, kDa) of predicted N-terminal mitochondrial targeting signal (MTS) when available, and the reliability class (RC) with classes from 1=maximal reliability to 5=minimal reliability.

Mitoprot II reported data consist in: percentage of mitochondrial localization (% mt), position (aa) of the cleavage site and Mw (kDa) of predicted MTS when available. n.p.: not predictable. Note that the two employed software often produce predictions with differing probabilities.

Supplementary Table S2: List of primers used for cloning

**Primers List**	
**For cloning into pCR4blunt TOPO vector, flanking T7 promoter**	
F_UHV1-NP	5’-ATGGCTGCACTACAAAGAGCAGCCGTC-3’
F_wtUGV1-NP	5’-ATGGCTGCCCTCCAGAGAGCTGCTGTCAAT-3’
F_mutUGV1-NP	5’-ATGGCTGCCCTCCAGGAGGCCGCCGTCAA-3’
F_HISV1-NP	5’-ATGTCCTTGAACAAGGACCTTGC-3’
F_wtJUNV-NP	5’-ATGGCACACTCCAAAGAGGTTCCAAGCTTT-3’
F_LCMV-NP	5’-ATGTCCTTGTCTAAGGAAGTTAAGAGCTTCCAATG-3’
R_HA	5’-TCAGGCATAATCTGGGACATCATAAGGGTAC-3’
R_UGV1-NP	5’-TTAGGCTGTAGCTGGTGTGGGATCCTTGATCTTC-3’
**For cloning into pGEM4Z vector, flanking T7 promoter**	
F_HISV1-NP_HindIII	5’-ttgcagcgAAGCTTATGTCCTTGAACAAGGACC-3’
R_HA_BamHI	5’-gatcagttGGATCCTCAGGCATAATCTGGGACAT-3’
**For cloning into pGEM4Z vector, flanking SP6 promoter**	
F_wtUGV1-NP_KpnI	5’-tttcataaGGTACCATGGCTGCCCTCCAGAGAGC-3’
F_mutUGV1-NP_KpnI	5’-tttcataaGGTACCATGGCTGCCCTCCAGGAGG-3’
R_UGV1-NP_SalI	5’-tttcaacgGTCGACTTAGGCTGTAGCTGGTGTGG-3’
F_UHV1-NP_BamHI	5’-tttcaggaGGATCCATGGCTGCACTACAAAGAGC-3’
R_UHV1-NP_PstI	5’-tttcatatCTGCAGTTAGACCTCCACAGGCCCAG-3’
F_HISV1-NP_BamHI	5’-ttagatcgGGATCCATGTCCTTGAACAAGGACCT-3’
R_HISV1-NP_HindIII	5’-tggccgcgAAGCTTTTAGTTGTTCATTATGTAGTTG-3’
F_wtJUNV-NP_EcoRI	5’-tttcaccaGAATTCATGGCACACTCCAAAGAGGT-3’
R_wtJUNV-NP_SalI	5’-tatctaatGTCGACTCACAGTGCATAGGCTGCCT-3’
F_LCMV-NP_EcoRI	5’-ttgcgccaGAATTCATGTCCTTGTCTAAGGAAGT-3’
R_LCMV-NP_SalI	5’-tttcacagGTCGACTCAGAGTGTCACAACATTTG-3’

Supplementary Table S3: List of antibodies used for immunoblot, IF and immuno-EM

**Antibodies used for immunoblot**		
Primary antibodies	**host**	**company and dilution**
anti-α-tubulin	mouse	Calbiochem CP06, 1:500
anti-mitochondrial outer membrane translocase 20 (TOM20)	rabbit	Santa Cruz sc-11415, 1:1000
anti-voltage-dependent anion-channel (VDAC)	mouse	Abcam ab14734, 1:500
anti-mitochondrial ribosomal protein S35 (MRPS35)	rabbit	Proteintech 16457-1-AP, 1:500 or 1:1000
anti-mitofusin 2 (MFN2)	mouse	Abcam ab56889, 1:100 or 1:200
anti-FLAG	mouse	Flarebio CSB-MA000021M0m, 1:500
anti-HA	mouse	Sigma H3663, 1:500
affinity-purified anti-UHV-NP[27]	rabbit	custom made, 1:500
anti-MTS-NP	rabbit	custom made, 1:200
anti-hartmanivirus NP (anti-Hartmani-NP)	rabbit	custom made, 1:500
Secondary antibodies	**host**	**company and dilution**
IRDye 800CW Donkey anti-rabbit	donkey	LI-COR Biosciences, 1:10000
IRDye 680RD Donkey anti-mouse	donkey	LI-COR Biosciences, 1:10000
**Antibodies used for IF**		
Primary antibodies	**host**	**company and dilution**
anti-mitochondria (clone MTCO2)	mouse	Abcam ab3298, 1:1000
anti-FLAG	mouse	Flarebio CSB-MA000021M0m, 1:1000
anti-FLAG	rabbit	Flarebio CSB-PA000337, 1:1000
anti-HA	mouse	Sigma H3663, 1:1000
anti-HA	rabbit	Flarebio CSB-PA275079, 1:1000
anti-MTS-NP	rabbit	custom made, 1:200
Secondary antibodies	**host**	**company and dilution**
Alexa Fluor 594 goat anti-rabbit	goat	Thermo Fisher Scientific, 1:400
Alexa Fluor 488 goat anti-mouse	goat	Thermo Fisher Scientific, 1:500
**Antibodies used for immuno-EM**		
Primary antibodies	**host**	**company and dilution**
anti-pan reptarenavirus NP[30]	rabbit	custom made, 1:1000
Secondary antibodies	**host**	**company and dilution**

18 nm gold-conjugated goat anti-rabbit IgG antibody	goat	Milan Analytica AG, Rheinfelden, Switzerland, 1:20

Supplementary Figures and Legends

Supplementary Figure S1

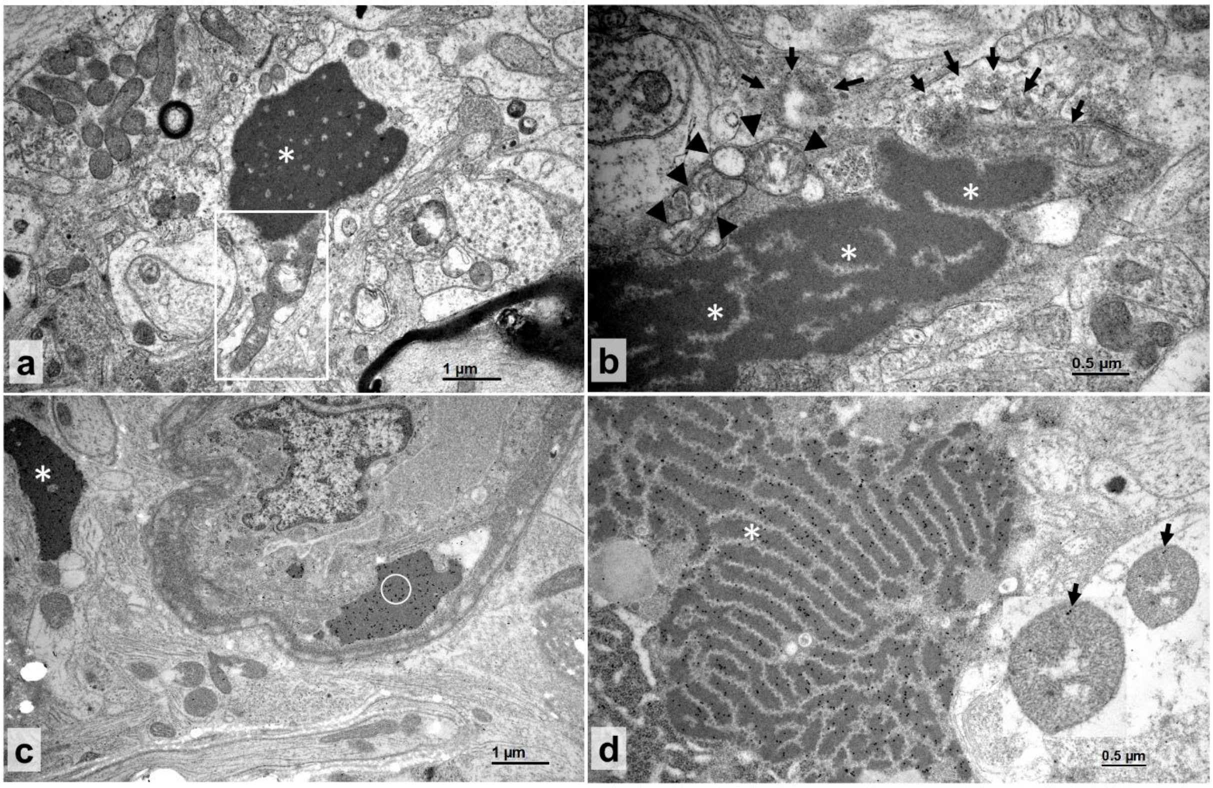

Supplementary Figure S1: TEM and immuno-EM of a BIBD-positive *B. constrictor* brain. **(a, b)** TEM, neurons. **(a)** Mitochondrion with vacuolated dissolved matrix (rectangle), adjacent to a larger IB (asterisk). **(b)** Large cytoplasmic IB (asterisks) with adjacent vacuolated mitochondria (arrowheads) and mitochondria with granular, dissolved matrix (arrows). **(c, d)** Immunogold labelling. **(c)** NP-positive electron-dense cytoplasmic IBs (asterisk) within a neuron adjacent to a small vessel with an IB (circle) in an endothelial cell. **(d)** NP-positive, less electron-dense irregular cytoplasmic IB (asterisk) and focal positive reaction within mitochondria (arrows).

Supplementary Figure S2

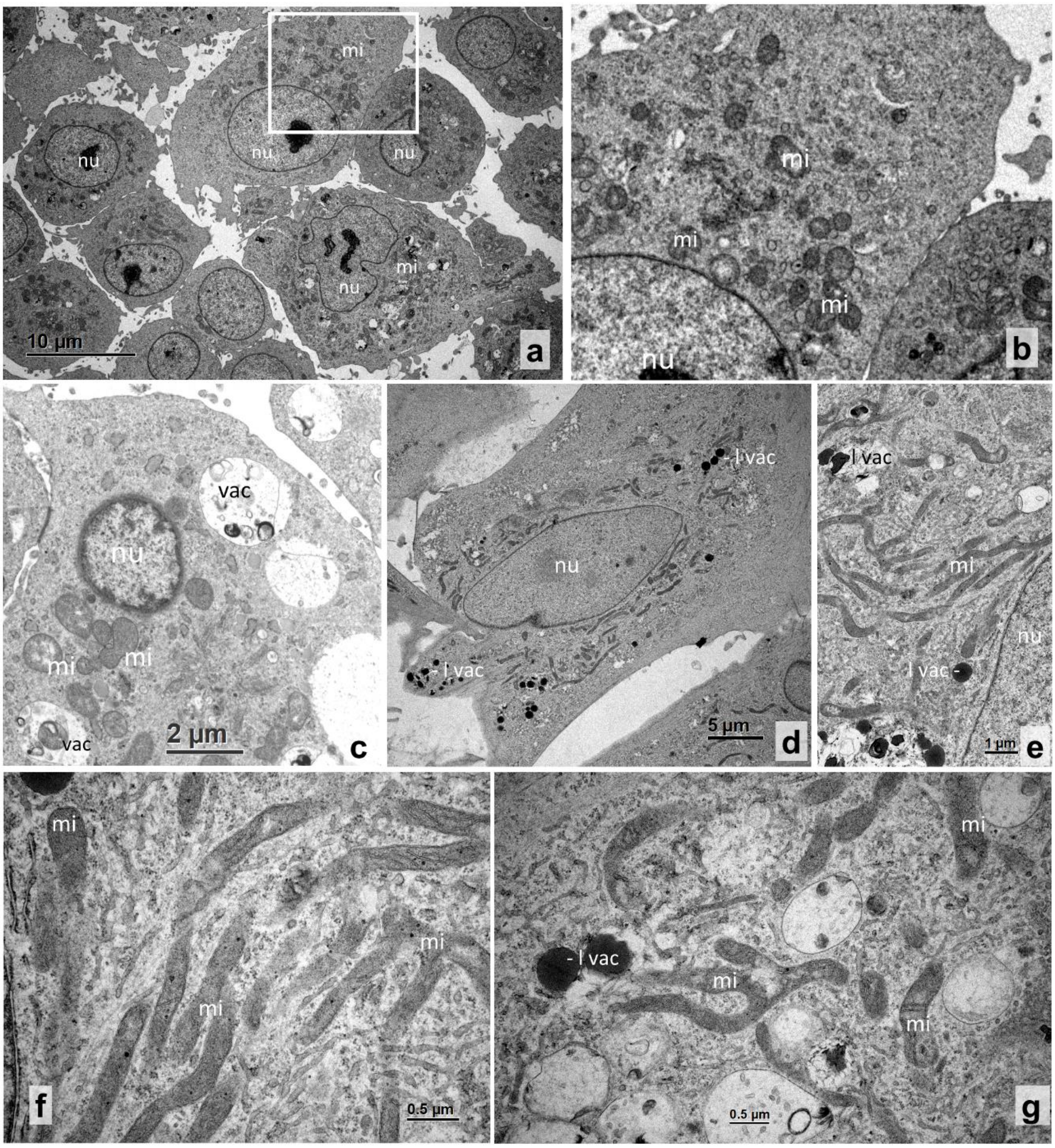

Supplementary Figure S2: TEM of mock (uninfected/untransfected) controls from different *B. constrictor* cell lines. (a-c) Overview of a tissue culture pellet from a kidney-derived cell line (I/1Ki) with regular mitochondrial arrangement (mi) and cytoplasmic vacuole (vac). **(b)** Higher magnification of the regular mitochondria (mi) shown in the white rectangle in **(a)**. **(d-g)** Overview of a tissue culture pellet from a brain-derived cell line (V/4Br) with regular mitochondrial arrangement (mi) and cytoplasmic lipid vacuole (l vac). nu: nucleus.

Supplementary Figure S3

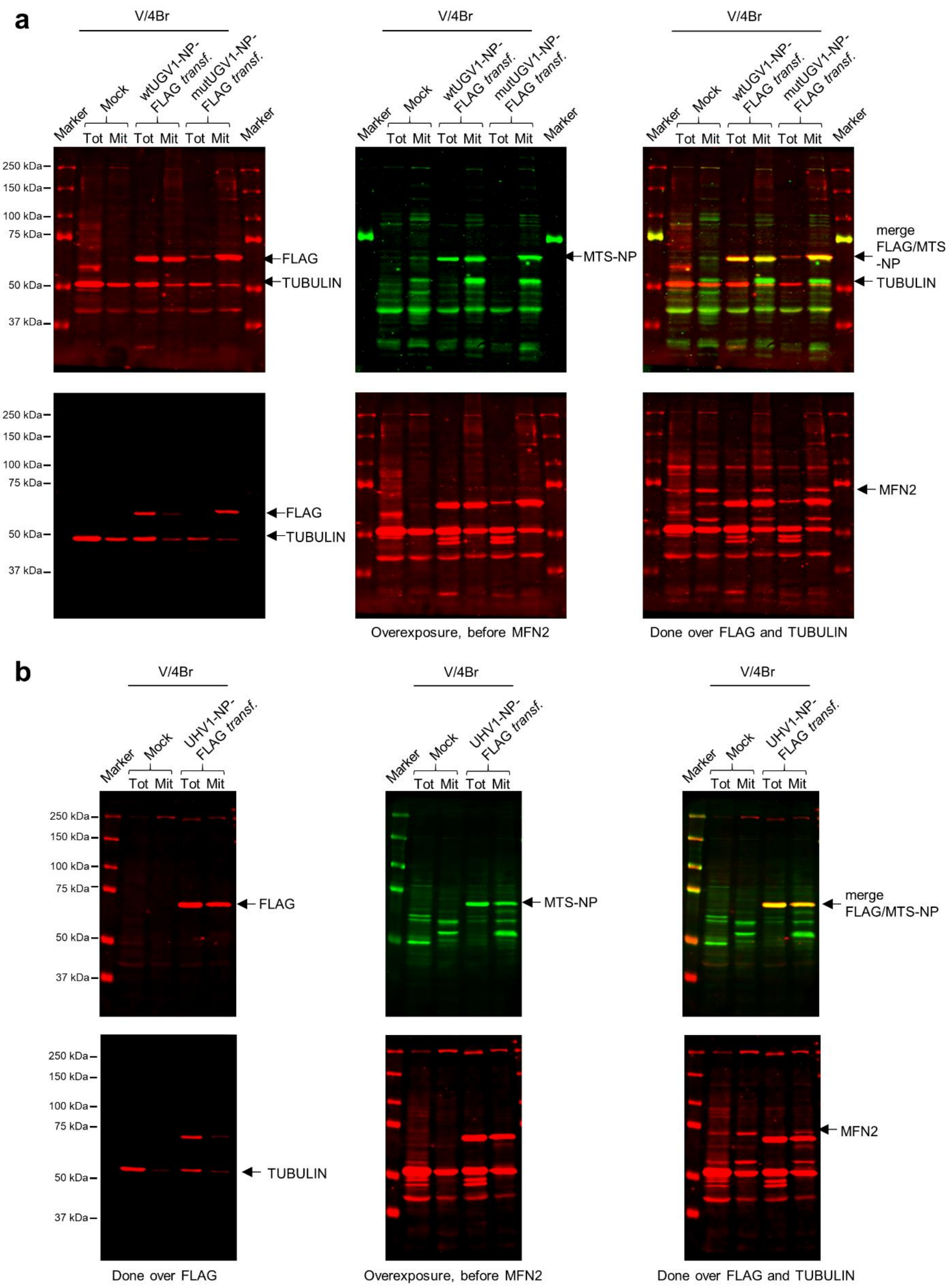

c

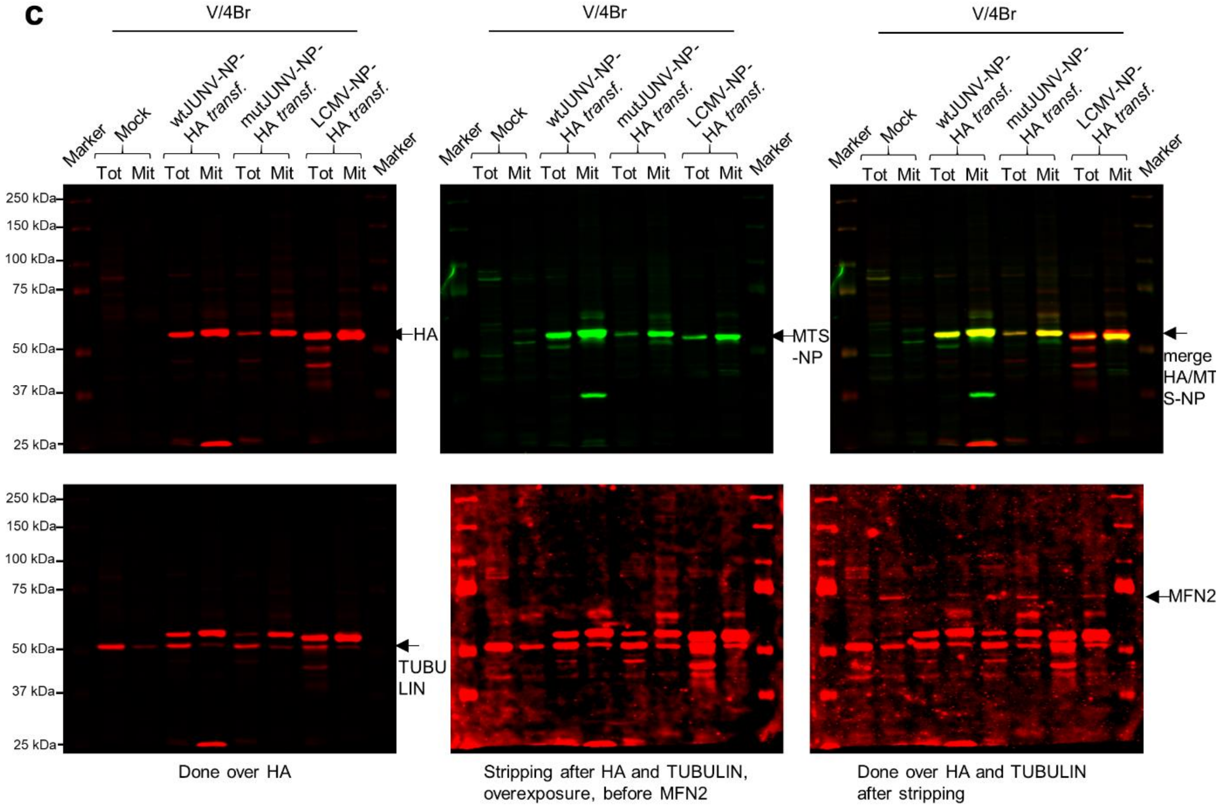

V/4Br
Marker
Mock
wtJUNV-NP-HA transf.
mutJUNV-NP-HA transf.
LCMV-NP-HA transf.
Tot Mit Tot Mit Tot Mit Tot Mit
250 kDa
150 kDa
100 kDa
75 kDa
50 kDa
37 kDa
25 kDa
HA
MTS-NP
merge HA/MTS-NP
TUBULIN
MFN2
Done over HA
Stripping after HA and TUBULIN, overexposure, before MFN2
Done over HA and TUBULIN after stripping

d

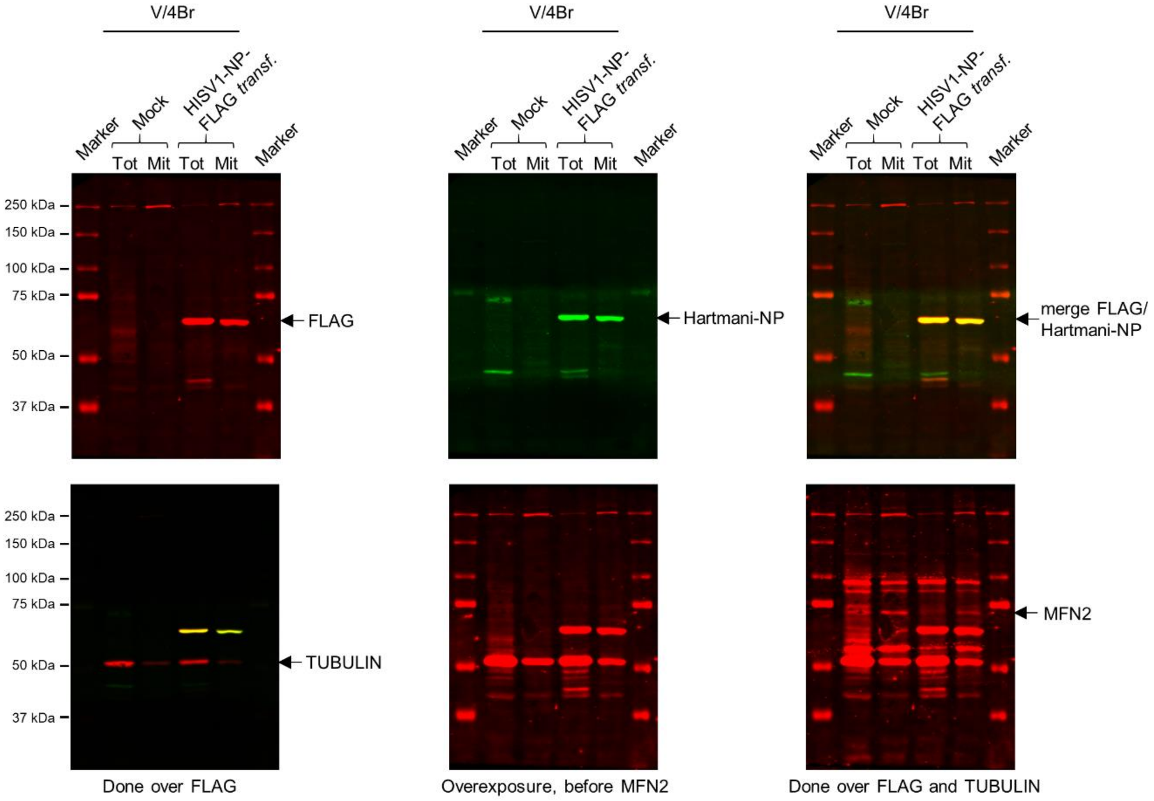

V/4Br
Marker
Mock
HISV1-NP-FLAG transf.
Tot Mit Tot Mit
250 kDa
150 kDa
100 kDa
75 kDa
50 kDa
37 kDa
FLAG
Hartmani-NP
merge FLAG/Hartmani-NP
TUBULIN
MFN2
Done over FLAG
Overexposure, before MFN2
Done over FLAG and TUBULIN

Supplementary Figure S3: Full-length blots from boid cells transfected with different arenaviral NPs. (a-d) Full-length/uncropped versions of the immunoblots presented in Figure 3, panels a-d, of whole-cell lysates (Tot) and mitochondrial preparations (Mit) from *Boa constrictor* V/4Br cells transfected with constructs expressing wt or mutUGV1-NP-FLAG **(a)**, UHV1-NP-FLAG **(b)**, wt or mutJUNV-NP-HA, or LCMV-NP-HA **(c)** and HISV1-NP-FLAG **(d)**, all at three dpt. Non-transfected (Mock) samples were used as negative controls. About 1/5 of the transfected cells were used for whole-cell lysate procedure (Tot) and the other 4/5 for mitochondria isolation (Mit). 20 µg **(a, b)** or 12 µg **(c, d)** of protein per sample from both Tot and Mit were loaded on standard SDS-PAGE gels followed by immunoblotting analyses. The nitrocellulose membranes were incubated sequentially with the following antibodies in the presented order: 1) mouse anti-FLAG tag 1:500 **(a, b, d)** or mouse anti-HA tag 1:500 **(c)**; 2) rabbit anti-MTS-NP 1:200 **(a, b, c)** or rabbit anti-Hartmani-NP 1:500 **(d)**; 3) mouse anti-tubulin 1:500 **(a-d)**; 4) mouse anti-MFN2 1:200 **(a, b, d)** or 1:100 **(c)**. Anti-tubulin and anti-MFN2 specific signals at known molecular weight did not require membrane stripping, except for panel c, where a stripping step was introduced before probing with anti-MFN2. **(a, b)** FLAG tag, tubulin and MFN2 in red (secondary antibody: IRDye 680RD Donkey anti-mouse); MTS-NP in green (secondary antibody: IRDye 800CW Donkey anti-rabbit). **(c)** HA tag, tubulin and MFN2 in red (secondary antibody: IRDye 680RD Donkey anti-mouse); MTS-NP in green (secondary antibody: IRDye 800CW Donkey anti-rabbit). **(d)** FLAG tag, tubulin and MFN2 in red (secondary antibody: IRDye 680RD Donkey anti-mouse); Hartmani-NP in green (secondary antibody: IRDye 800CW Donkey anti-rabbit). Immunodetection was performed using the Odyssey Infrared Imaging System (LICOR, Biosciences) providing also the molecular marker (Precision Plus Protein Dual Color Standards, Bio-Rad) used.

Supplementary Figure S4

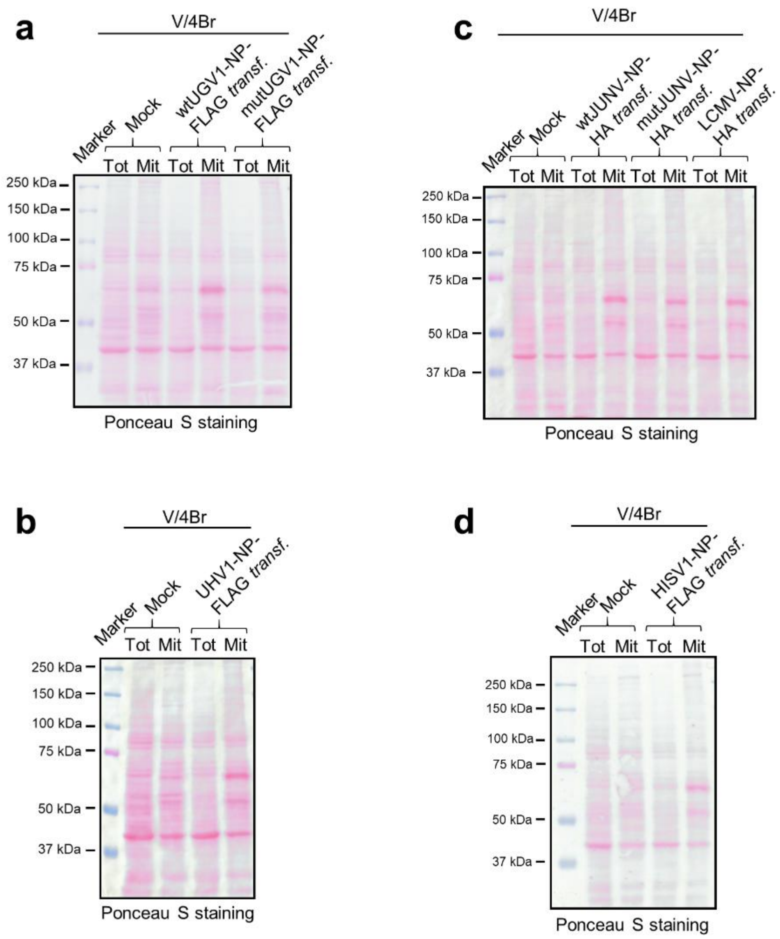

Supplementary Figure S4: Ponceau S stainings of immunoblots from boid cells transfected with different arenaviral NPs. (a-d) Ponceau S stainings of nitrocellulose membranes, representing a reference for loading for immunoblotting analyses reported in Figure 3, panels a-d. Whole-cell lysates (Tot) and mitochondrial preparations (Mit) were obtained from *Boa constrictor* V/4Br cells transfected with constructs expressing wt or mutUGV1-NP-FLAG **(a)**, UHV1-NP-FLAG **(b)**, wt or mutJUNV-NP-HA, or LCMV-NP-HA **(c)** and HISV1-NP-FLAG **(d)**, all at three dpt. Non-transfected (Mock) samples were used as negative controls. 20 µg **(a, b)** or 12 µg **(c, d)** of protein per sample were loaded on standard SDS-PAGE gels followed by immunoblotting analyses. Molecular weight marker: Precision Plus Protein Dual Color Standards (Bio-Rad).

Supplementary Figure S5

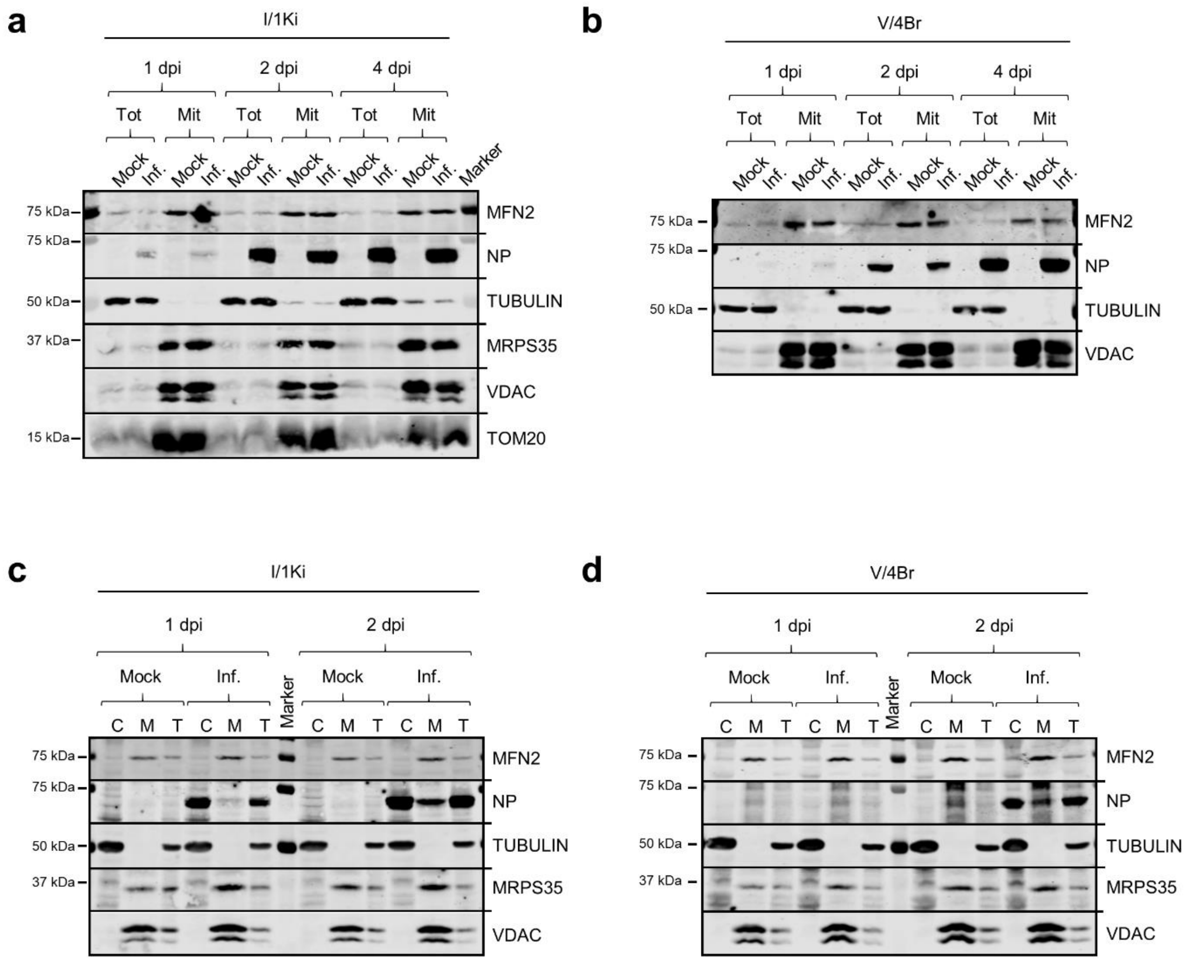

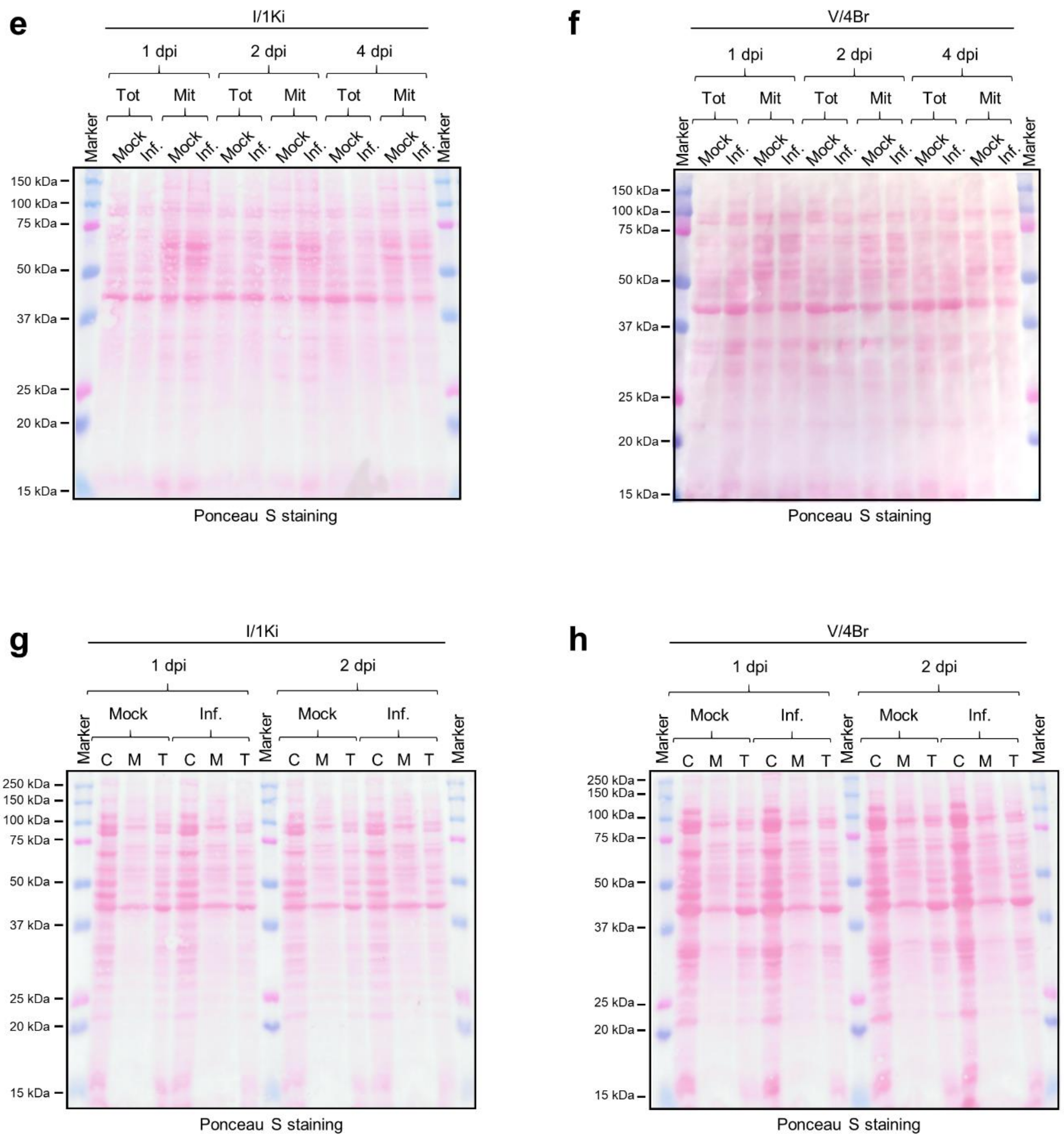

Supplementary Figure S5: Subcellular analyses of reptarenaviral NP in infected boid cells. (a, b) Immunoblot analyses of whole-cell lysates (Tot) and mitochondrial preparations (Mit) obtained from reptarenavirus-infected *Boa constrictor* I/1Ki **(a)** and V/4Br **(b)** cells at one, two and four dpi. Uninfected (Mock) samples provide the negative controls. About 1/5 of the transfected cells were used for whole-cell lysate procedure (Tot) and the other 4/5 for mitochondria isolation (Mit). 15 **(a)** and 25 µg **(b)** of protein per sample were separated on SDS-PAGE followed by immunoblotting to detect tubulin (a cytosolic marker), TOM20,

VDAC, MRPS35 and MFN2 (mitochondrial markers) and reptarenaviral NP. **(c, d)** Subcellular fractionation analyses of reptarenavirus-infected *Boa constrictor* I/1Ki **(c)** and V/4Br **(d)** cells at one and two dpi. Uninfected (Mock) samples provide the negative controls. Cytosolic (C) and mitochondrial (M) fractions and total cellular extracts (T) were obtained from a standard kit-based (Abcam, ab109719) cell fractionation procedure. Samples of intact cells (T) were collected before mixing the cell suspension with detergent solutions used for cell fractionation. Before SDS-PAGE, the cytosolic and mitochondrial fractions were 10-fold concentrated using Amicon Ultra-0.5 Centrifugal Filter Units (Merck Millipore) following the manufacturer's instructions. The fractions were brought to equal volumes, and 15 µl of each fraction (C, M and T) were loaded and separated in SDS-PAGE gels, followed by immunoblotting. Antibodies detecting tubulin (a cytosolic marker), VDAC, MRPS35 and MFN2 (mitochondrial markers), and reptarenaviral NP were used.

All four nitrocellulose membranes were cut into upper, middle and lower parts, that were subsequently incubated with antibodies against: upper+middle part (before cut), mouse anti-tubulin 1:500 (secondary antibody: IRDye 680RD Donkey anti-mouse); upper part, rabbit affinity-purified anti-UHV-NP 1:500 (secondary antibody: IRDye 800CW Donkey anti-rabbit), followed by mouse anti-MFN2 1:200 (secondary antibody: IRDye 680RD Donkey anti-mouse); middle part, mouse anti-VDAC 1:500 (secondary antibody: IRDye 680RD Donkey anti-mouse) followed by rabbit anti-MRPS35 (for panels a, c and d**)** 1:500 (secondary antibody: IRDye 800CW Donkey anti-rabbit); lower part, rabbit anti-TOM20 1:1000 (only for panel a) (secondary antibody: IRDye 800CW Donkey anti-rabbit). Immunodetections were performed using the Odyssey Infrared Imaging System (LICOR, Biosciences). **(e-h)** Ponceau S staining of nitrocellulose membranes, representing a reference for loading for immunoblotting analyses reported in Figure S5, panels a-d. Membranes from analyses of Tot and Mit from reptarenavirus-infected I/1Ki **(e)** and V/4Br **(f)** and from kit-based subcellular fractionation analyses of reptarenavirus-infected I/1Ki **(g)** and V/4Br **(h)** cells are shown. Note that for the

second approach, the gel loading is based on equal volumes and therefore the protein amount loaded varies considerably between the fractions **(g, h)**. Molecular weight marker: Precision Plus Protein Dual Color Standards (Bio-Rad).

Supplementary Figure S6

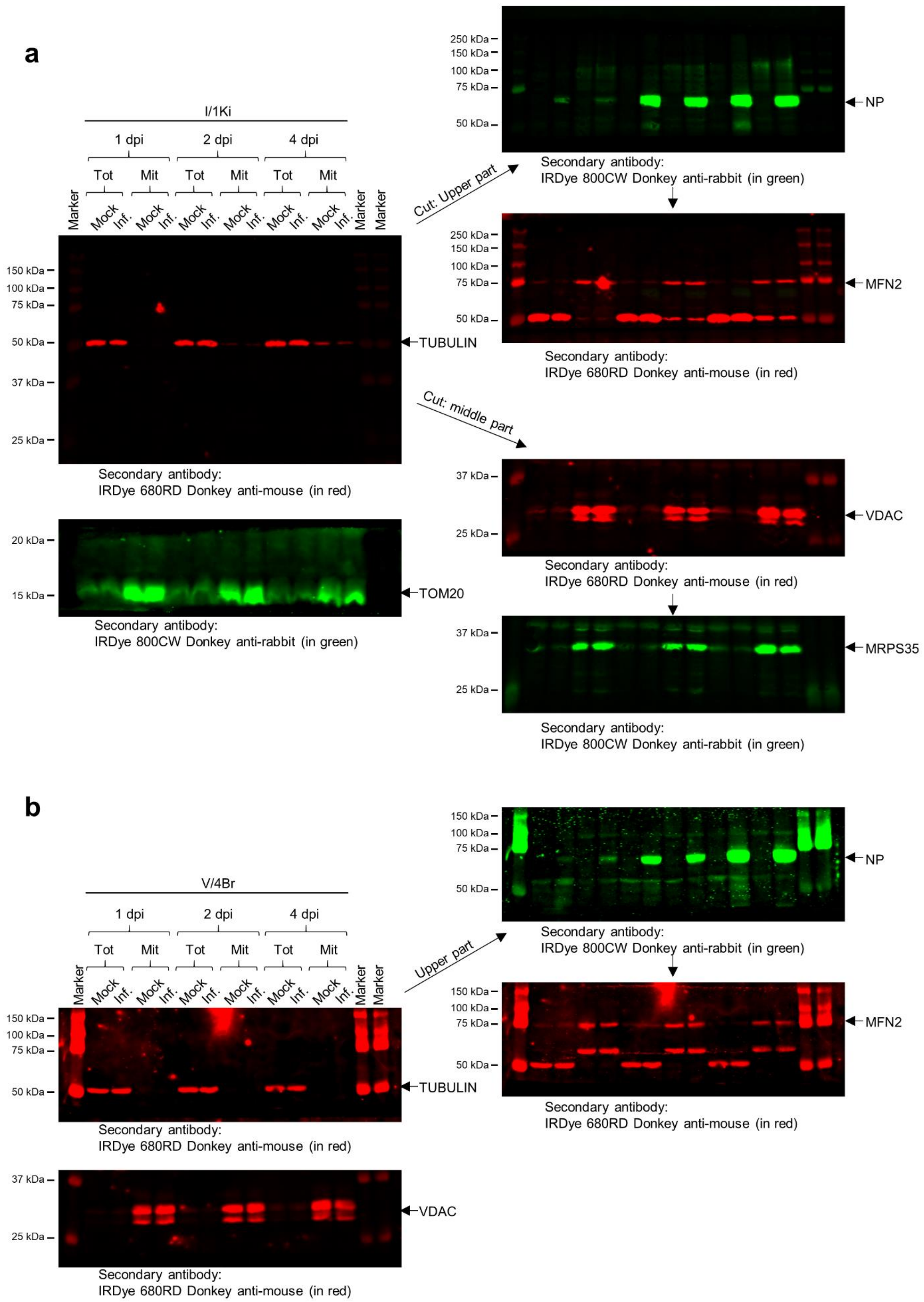

c

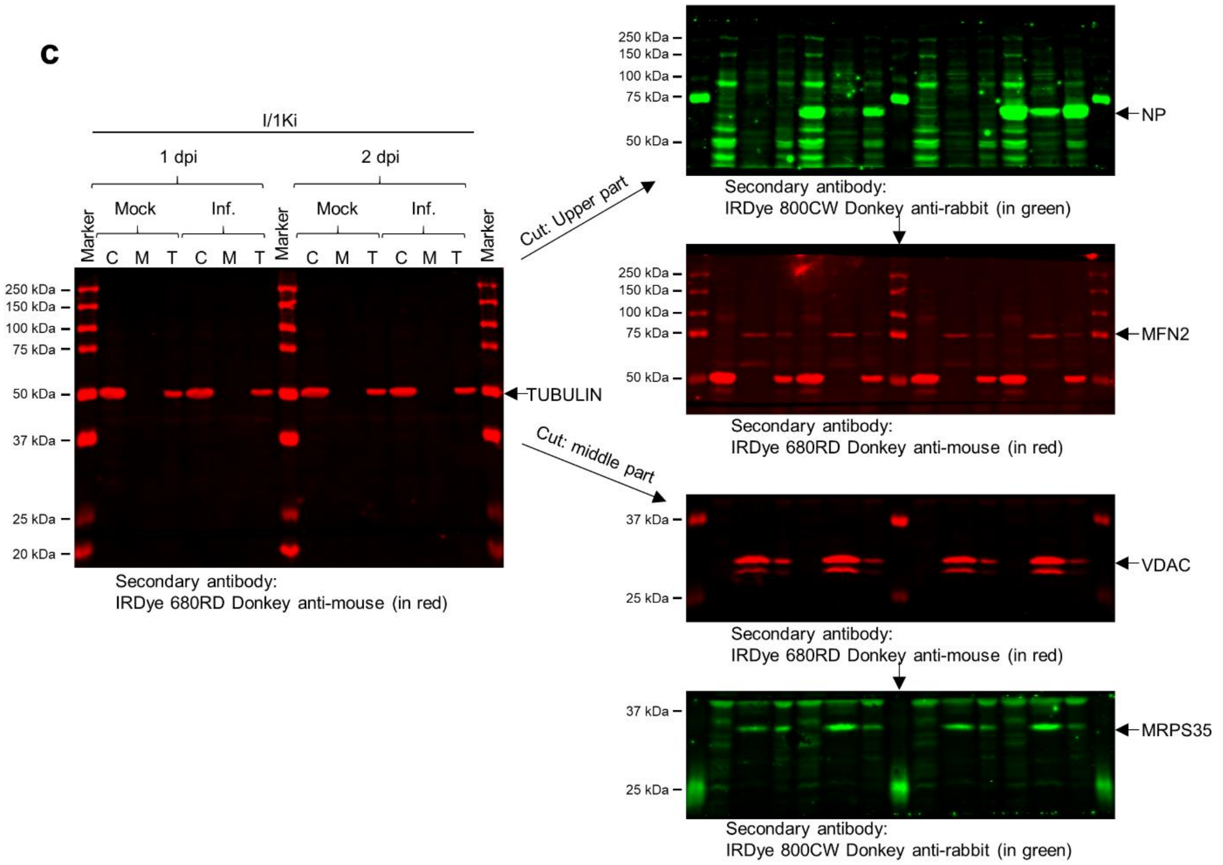
I/1Ki
1 dpi
2 dpi
Mock
Inf.
Marker
C M T C M T
250 kDa
150 kDa
100 kDa
75 kDa
50 kDa
37 kDa
25 kDa
20 kDa
TUBULIN
Secondary antibody:
IRDye 680RD Donkey anti-mouse (in red)
Cut: Upper part
Cut: middle part
NP
Secondary antibody:
IRDye 800CW Donkey anti-rabbit (in green)
MFN2
Secondary antibody:
IRDye 680RD Donkey anti-mouse (in red)
VDAC
Secondary antibody:
IRDye 680RD Donkey anti-mouse (in red)
MRPS35
Secondary antibody:
IRDye 800CW Donkey anti-rabbit (in green)

d

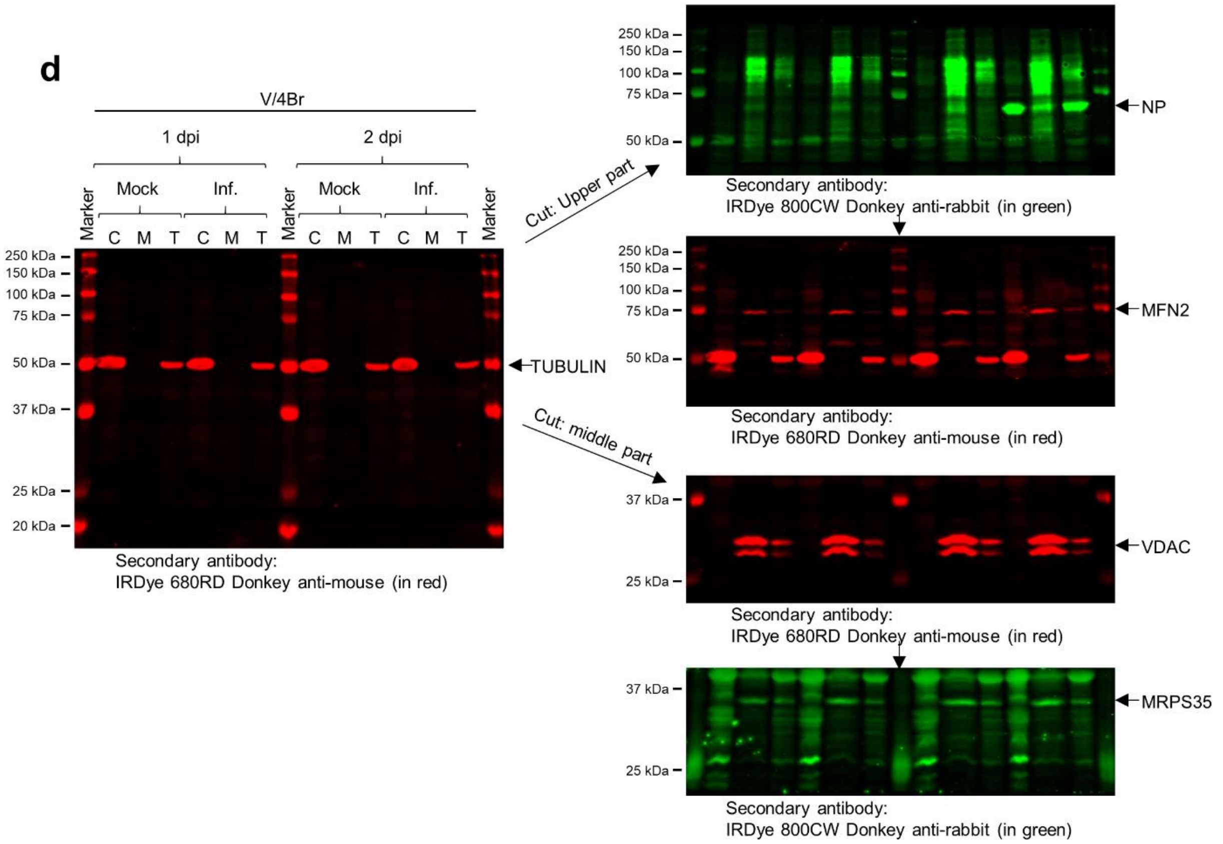
V/4Br
1 dpi
2 dpi
Mock
Inf.
Marker
C M T C M T
250 kDa
150 kDa
100 kDa
75 kDa
50 kDa
37 kDa
25 kDa
20 kDa
TUBULIN
Secondary antibody:
IRDye 680RD Donkey anti-mouse (in red)
Cut: Upper part
Cut: middle part
NP
Secondary antibody:
IRDye 800CW Donkey anti-rabbit (in green)
MFN2
Secondary antibody:
IRDye 680RD Donkey anti-mouse (in red)
VDAC
Secondary antibody:
IRDye 680RD Donkey anti-mouse (in red)
MRPS35
Secondary antibody:
IRDye 800CW Donkey anti-rabbit (in green)

Supplementary Figure S6: Full-length blots from subcellular analyses of reptarenaviral NP in infected boid cells. (a, b) Full-length/uncropped versions of the immunoblot presented in Supplementary Figure S5, panels a and b, of whole-cell lysates (Tot) and mitochondrial preparations (Mit) obtained from reptarenavirus-infected *Boa constrictor* I/1Ki **(a)** and V/4Br **(b)** cells at one, two and four dpi. Uninfected (Mock) samples provide the negative controls. About 1/5 of the transfected cells were used for whole-cell lysate procedure (Tot) and the other 4/5 for mitochondria isolation (Mit). 15 **(a)** and 25 µg **(b)** of protein per sample were separated on SDS-PAGE followed by immunoblotting to detect tubulin (a cytosolic marker), TOM20, VDAC, MRPS35 and MFN2 (mitochondrial markers) and reptarenaviral NP. **(a)** The nitrocellulose membrane was cut into upper, middle and lower parts, that were subsequently incubated with antibodies against: upper+middle part (before cut), mouse anti-tubulin 1:500 (secondary antibody: IRDye 680RD Donkey anti-mouse); upper part, rabbit affinity-purified anti-UHV-NP 1:500 (secondary antibody: IRDye 800CW Donkey anti-rabbit), followed by mouse anti-MFN2 1:200 (secondary antibody: IRDye 680RD Donkey anti-mouse); middle part, mouse anti-VDAC 1:500 (secondary antibody: IRDye 680RD Donkey anti-mouse) followed by rabbit anti-MRPS35 1:500 (secondary antibody: IRDye 800CW Donkey anti-rabbit); lower part, rabbit anti-TOM20 1:1000 (secondary antibody: IRDye 800CW Donkey anti-rabbit). **(b)** The nitrocellulose membrane was cut into upper, middle and lower parts (lower part not considered), that were subsequently incubated with antibodies against: upper part, mouse anti-tubulin 1:500 (secondary antibody: IRDye 680RD Donkey anti-mouse), followed by rabbit affinity-purified anti-UHV-NP 1:500 (secondary antibody: IRDye 800CW Donkey anti-rabbit) and by mouse anti-MFN2 1:200 (secondary antibody: IRDye 680RD Donkey anti-mouse); middle part, mouse anti-VDAC 1:500 (secondary antibody: IRDye 680RD Donkey anti-mouse).

(c, d) Full-length/uncropped versions of the immunoblots presented in Supplementary Figure S5, panels c and d, of subcellular fractionation analyses of reptarenavirus-infected *Boa*

constrictor I/1Ki **(c)** and V/4Br **(d)** cells at one and two dpi. Uninfected (Mock) samples provide the negative controls. Cytosolic (C) and mitochondrial (M) fractions and total cellular extracts (T) were obtained from a standard kit-based (Abcam, ab109719) cell fractionation procedure. Before SDS-PAGE separation the fractions were brought to same volumes, and 15 µl of each fraction (C, M and T) were loaded and separated in SDS-PAGE gels, followed by immunoblotting. Note that for this experiment, the gel loading is based on equal volumes and therefore the protein amount loaded varies considerably between the fractions. Antibodies detecting tubulin (a cytosolic marker), VDAC, MRPS35 and MFN2 (mitochondrial markers), and reptarenaviral NP were used. The nitrocellulose membranes were cut into upper, middle and lower parts (lower parts not considered), that were subsequently incubated with antibodies against: upper+middle part (before cut), mouse anti- tubulin 1:500 (secondary antibody: IRDye 680RD Donkey anti-mouse); upper part, rabbit affinity-purified anti-UHV-NP 1:500 (secondary antibody: IRDye 800CW Donkey anti-rabbit), followed by mouse anti-MFN2 1:200 (secondary antibody: IRDye 680RD Donkey anti-mouse); middle part, mouse anti-VDAC 1:500 (secondary antibody: IRDye 680RD Donkey anti-mouse) followed by rabbit anti-MRPS35 1:500 (secondary antibody: IRDye 800CW Donkey anti-rabbit). Immunodetections were performed using the Odyssey Infrared Imaging System (LICOR, Biosciences). Molecular weight marker: Precision Plus Protein Dual Color Standards (Bio-Rad).

Supplementary Figure S7

a

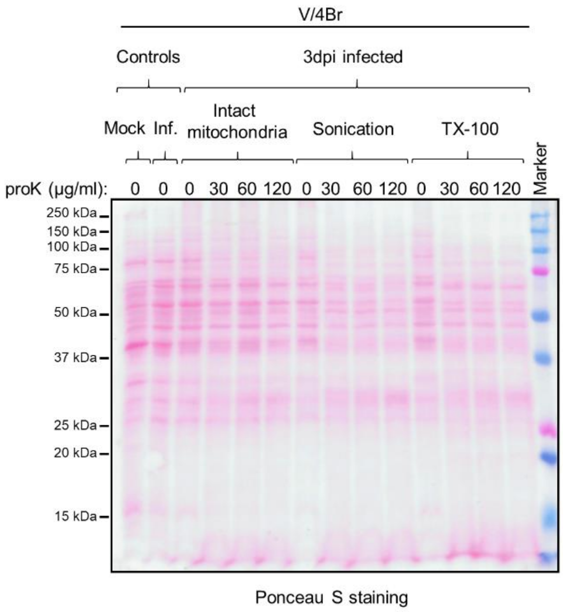

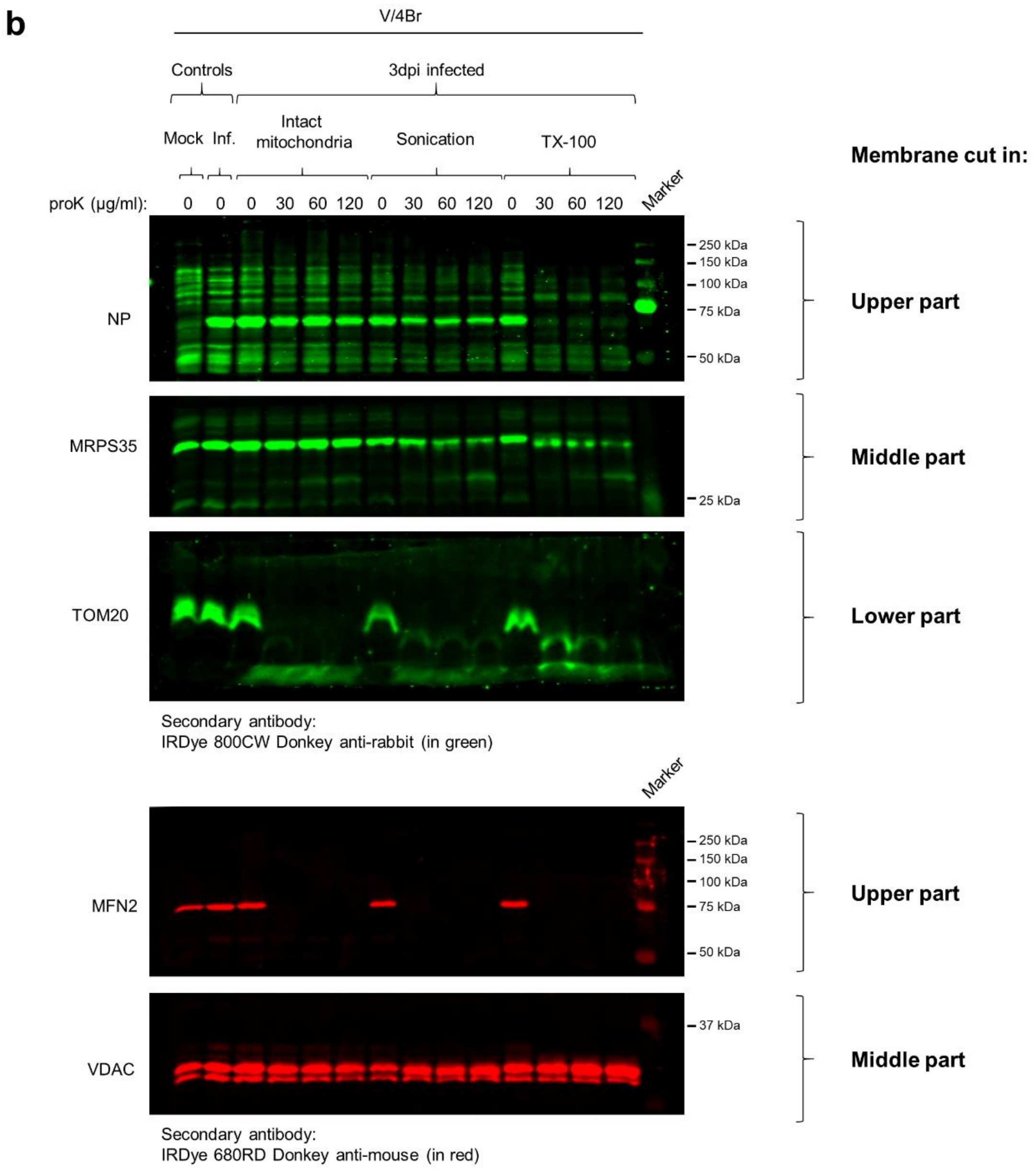

Supplementary Figure S7: Ponceau S staining and full-length blots of submitochondrial analyses of reptarenavirus NP in infected boid cells. (a) Ponceau S staining of the nitrocellulose membrane, representing a reference of proteolytic degradation for immunoblotting of the submitochondrial localization assay reported in Figure 4, panel a. Mitochondria were isolated at three dpi from reptarenavirus-inoculated *Boa constrictor* V/4Br cells and either treated directly with proK at 30, 60 or 120 µg/ml, or subjected to sonication or TX-100 lysis first, and then treated with proK. For each condition, a proK-untreated sample is

provided as control. **(b)** Full-length/uncropped versions of the immunoblot presented in Figure 4, panel a, of submitochondrial localization assay, determined by protease accessibility. Nitrocellulose membrane was cut into upper, middle and lower parts, that were subsequently incubated with antibodies against: upper part, rabbit affinity purified anti-UHV-NP 1:500 in green (secondary antibody: IRDye 800CW Donkey anti-rabbit), followed by mouse anti-MFN2 1:200 in red (secondary antibody: IRDye 680RD Donkey anti-mouse); middle part, rabbit anti-MRPS35 1:1000 in green (secondary antibody: IRDye 800CW Donkey anti-rabbit), followed by mouse anti-VDAC 1:500 in red (secondary antibody: IRDye 680RD Donkey anti-mouse); lower part, rabbit anti-TOM20 1:1000 in green (secondary antibody: IRDye 800CW Donkey anti-rabbit).

An uninfected (Mock) and a reptarenavirus-infected (Inf.) mitochondrial sample at three dpi serve respectively as negative and positive controls for the anti-UHV-NP antibody used to detect the reptarenaviral NP. The samples (25 µg/lane of protein derived from the mitochondrial fraction) were separated through standard SDS-PAGE followed by immunoblotting analyses. Immunodetections were performed using the Odyssey Infrared Imaging System (LICOR, Biosciences). Molecular weight marker: Precision Plus Protein Dual Color Standards (Bio-Rad).

Supplementary Figure S8

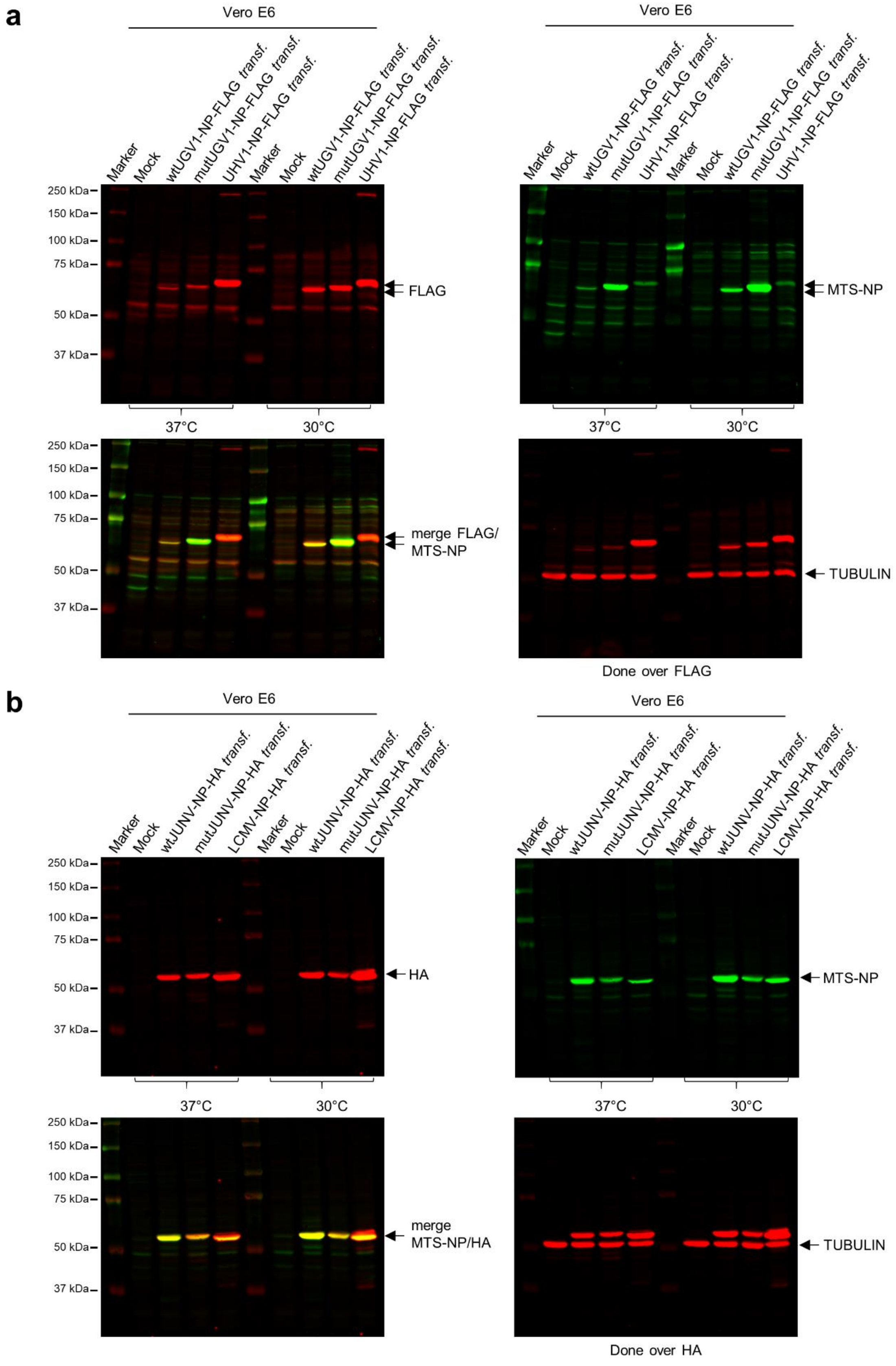

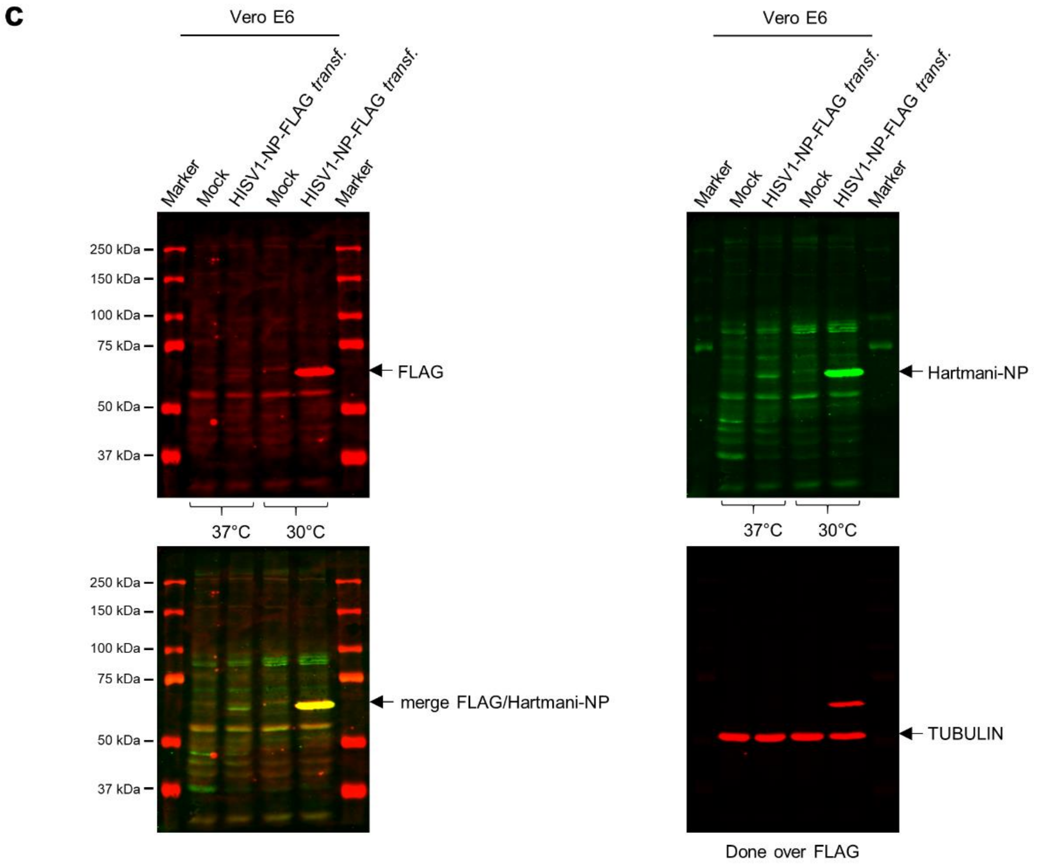

Supplementary Figure S8: Full-length blots from mammalian cells transfected with different arenaviral NPs. (a-c) Full-length/uncropped versions of the immunoblots presented in Figure 5, panels a-c, of whole-cell lysates obtained from monkey Vero E6 cells transfected with a construct expressing either wt or mutUGV1-NP-FLAG, or UHV1-NP-FLAG **(a)**, wt or mutJUNV-NP-HA, or LCMV-NP-HA **(b)**, HISV1-NP-FLAG **(c)**, at three dpt after incubation at either 37°C (left part) or 30°C (right part). Non-transfected (Mock) samples are provided as negative controls. 40 µg of protein per sample were loaded on standard SDS-PAGE gels, followed by immunoblotting analyses. The nitrocellulose membranes were incubated sequentially with the following antibodies in the presented order: 1) mouse anti-FLAG tag 1:500 **(a, c)** or mouse anti-HA tag 1:500 **(b)**; 2) rabbit anti-MTS-NP 1:200 **(a, b),** or rabbit anti-Hartmani-NP 1:500 **(c)**; 3) mouse anti-tubulin 1:500 **(a-c)**. Anti-tubulin specific signal at known molecular weight did not require membrane stripping. **(a)** FLAG tag and tubulin in red

(secondary antibody: IRDye 680RD Donkey anti-mouse); MTS-NP in green (secondary antibody: IRDye 800CW Donkey anti-rabbit). **(b)** HA tag and tubulin in red (secondary antibody: IRDye 680RD Donkey anti-mouse); MTS-NP in green (secondary antibody: IRDye 800CW Donkey anti-rabbit). **(c)** FLAG tag and tubulin in red (secondary antibody: IRDye 680RD Donkey anti-mouse); Hartmani-NP in green (secondary antibody: IRDye 800CW Donkey anti-rabbit). Immunodetection was performed using the Odyssey Infrared Imaging System (LICOR, Biosciences) providing also the molecular marker (Precision Plus Protein Dual Color Standards, Bio-Rad) used.

Supplementary Figure S9

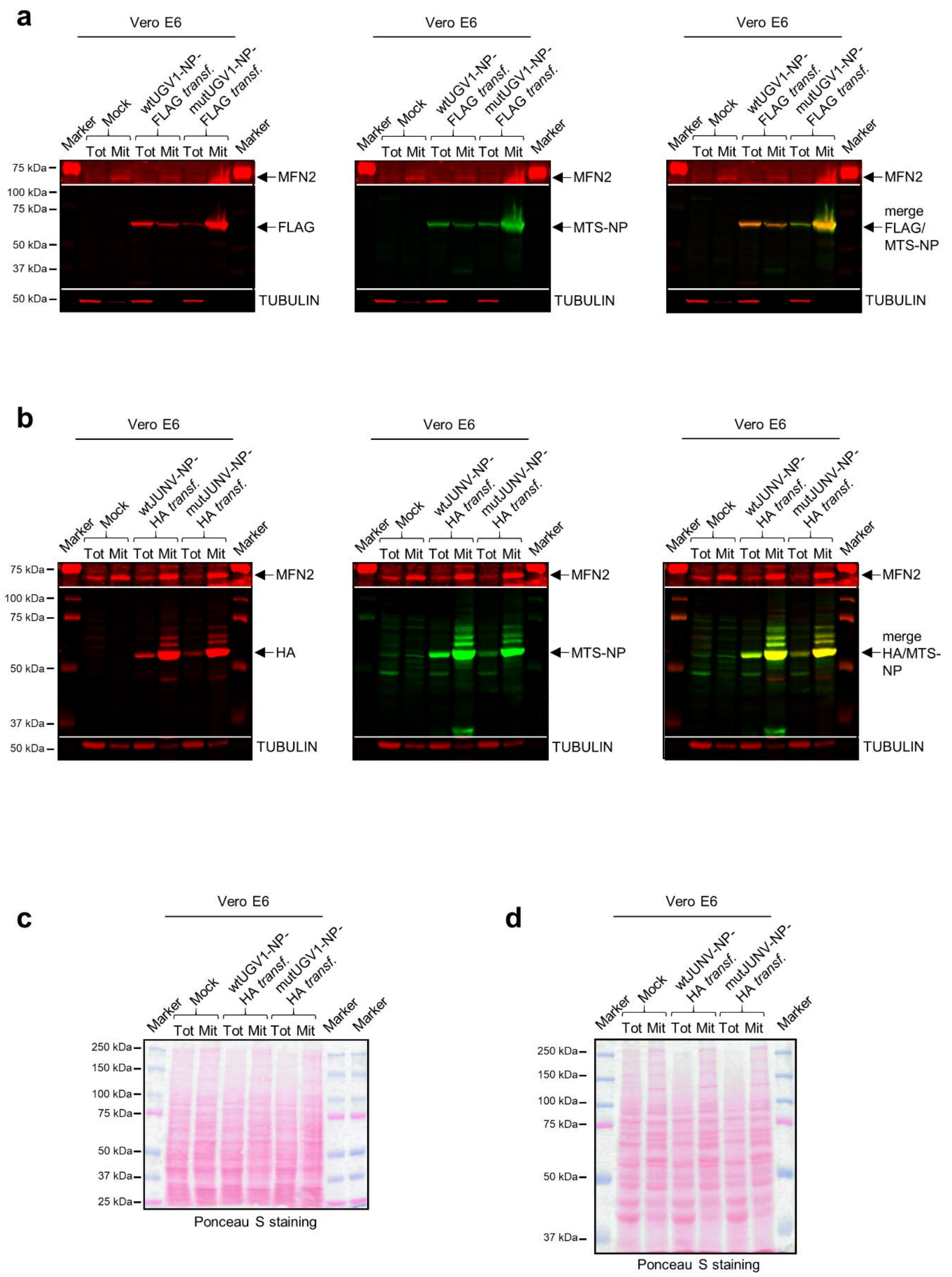

e

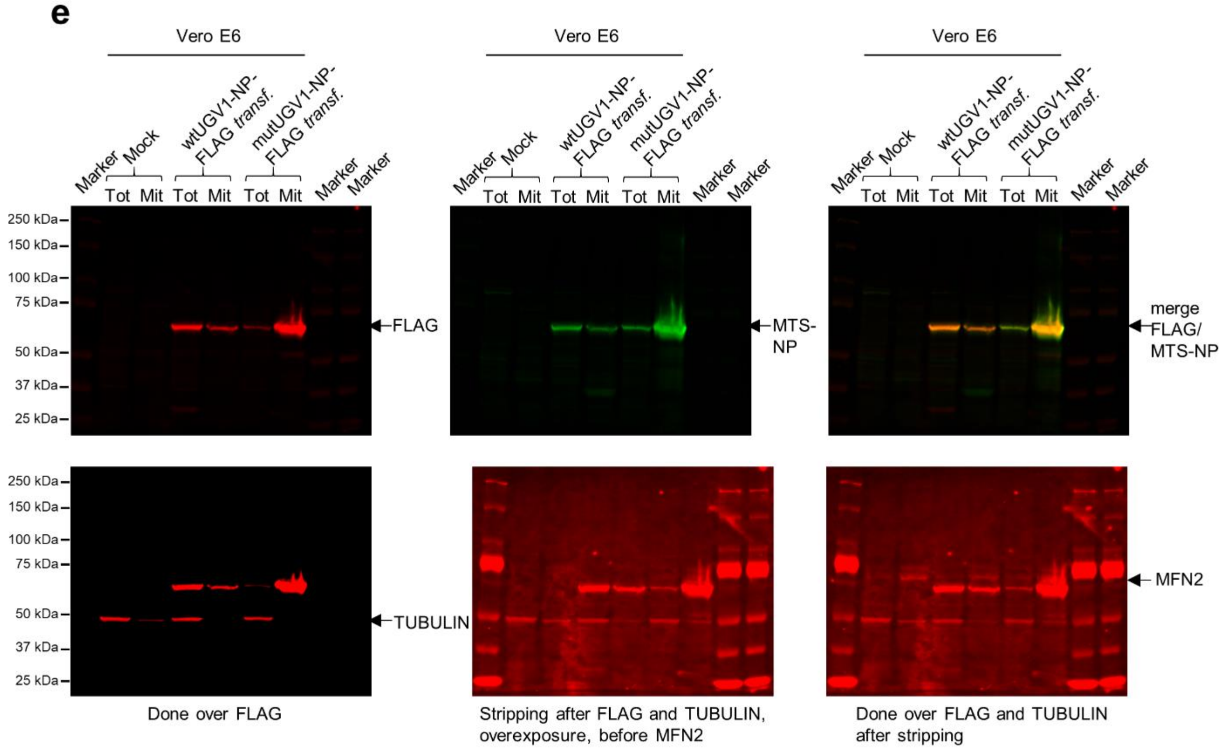
Vero E6
Marker
Mock
wtUGV1-NP-FLAG transf.
mutUGV1-NP-FLAG transf.
Tot Mit Tot Mit Tot Mit
250 kDa
150 kDa
100 kDa
75 kDa
50 kDa
37 kDa
25 kDa
FLAG
MTS-NP
merge FLAG/MTS-NP
TUBULIN
MFN2
Done over FLAG
Stripping after FLAG and TUBULIN, overexposure, before MFN2
Done over FLAG and TUBULIN after stripping

f

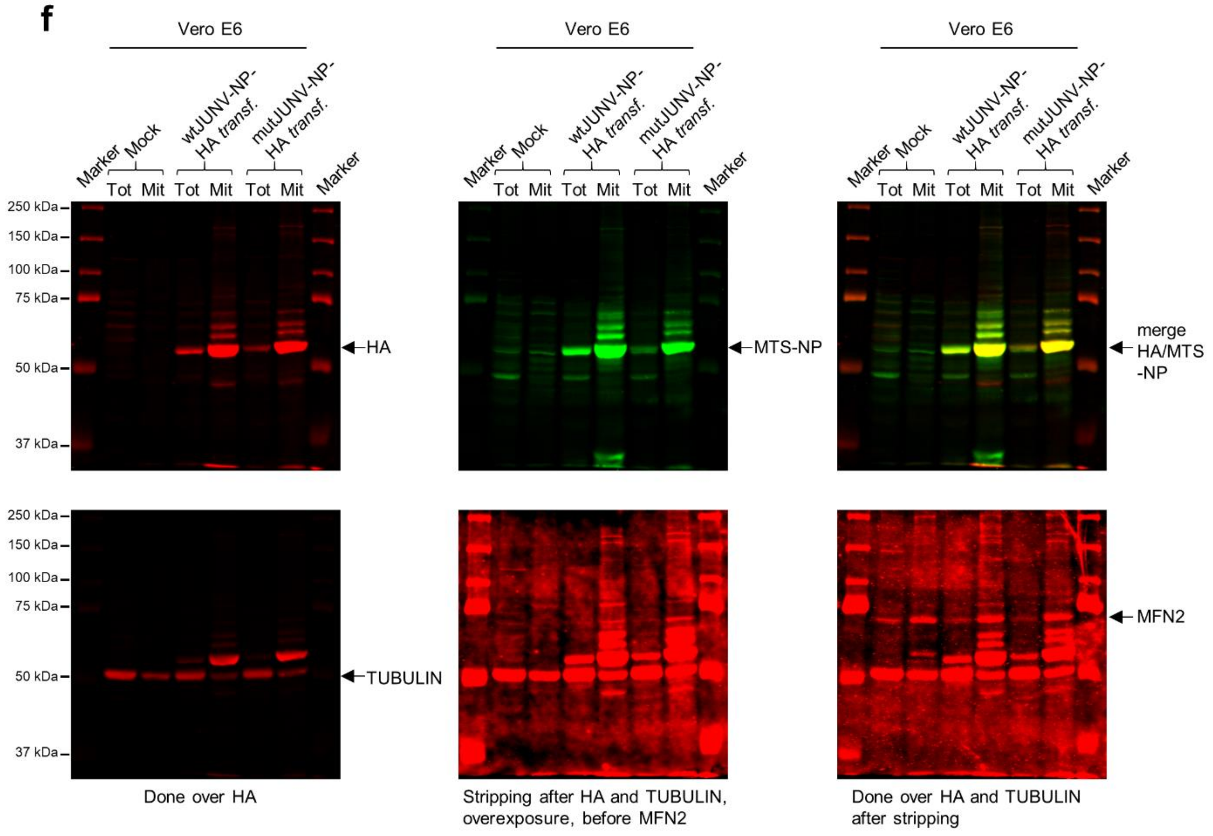
Vero E6
Marker
Mock
wtJUNV-NP-HA transf.
mutJUNV-NP-HA transf.
Tot Mit Tot Mit Tot Mit
250 kDa
150 kDa
100 kDa
75 kDa
50 kDa
37 kDa
HA
MTS-NP
merge HA/MTS-NP
TUBULIN
MFN2
Done over HA
Stripping after HA and TUBULIN, overexposure, before MFN2
Done over HA and TUBULIN after stripping

Supplementary Figure S9: Immunoblotting studies on mammalian cells transfected with arenavirus NPs. (a, b) Immunoblotting analyses of whole-cell lysates (Tot) and mitochondrial preparations (Mit) obtained from Vero E6 cells transfected with constructs expressing wt or mutUGV1-NP-FLAG **(a)**, or wt or mutJUNV-NP-HA **(b)**, at three dpt, at 37°C. Non-transfected (Mock) samples are provided as negative controls. About 1/5 of the transfected cells were used for whole-cell lysate procedure (Tot) and the other 4/5 for mitochondria isolation (Mit). 40 µg of protein per samples from both Tot and Mit were loaded on standard SDS-PAGE gels followed by immunoblotting analyses. The nitrocellulose membranes were incubated sequentially with the following antibodies in the presented order: 1) mouse anti-FLAG tag 1:500 **(a)** or mouse anti-HA tag 1:500 **(b)**; 2) rabbit anti-MTS-NP 1:200 **(a, b)**; 3) mouse anti-tubulin 1:500 **(a, b)**; 4) mouse anti-MFN2 1:100 **(a, b)**. A single stripping step was introduced before anti-MFN2 incubation. Tubulin and MFN2 (both in red, secondary antibody: IRDye 680RD Donkey anti-mouse) were used as cytosolic and mitochondrial marker, respectively. Arenavirus NPs (63-68 kDa) are indicated (black arrows). Left panels: FLAG **(a)** or HA tag **(b)** in red (secondary antibody: IRDye 680RD Donkey anti-mouse); middle panels: MTS-NP in green (secondary antibody: IRDye 800CW Donkey anti-rabbit); right panels: merged image. **(c, d)** Ponceau S stainings of nitrocellulose membranes, representing a reference for loading for immunoblotting analyses reported in panel a **(c)** and panel b **(d)**. **(e, f)** Full-length/uncropped versions of the immunoblots presented in panel a **(e)** and panel b **(f)**. Immunodetection was performed using the Odyssey Infrared Imaging System (LICOR, Biosciences) providing also the molecular marker (Precision Plus Protein Dual Color Standards, Bio-Rad) used.

Supplementary Figure S10

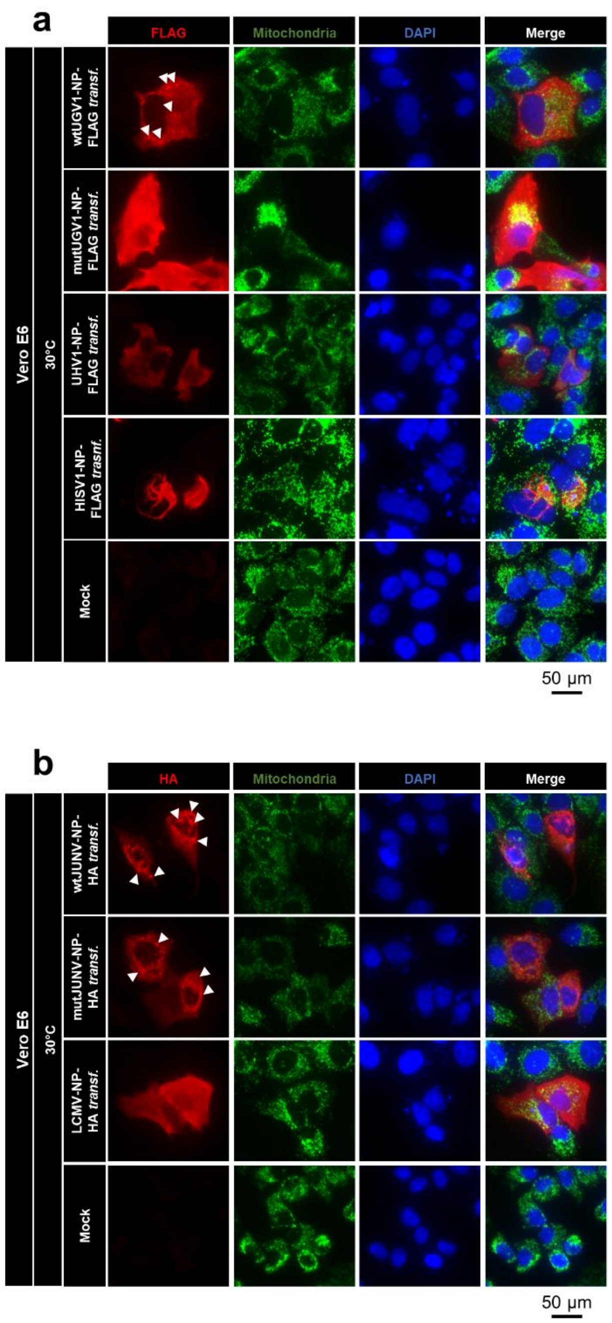

Supplementary Figure S10: IF studies on arenaviral NPs in mammalian cells. (a, b) Double IF images of monkey Vero E6 cells transfected with a construct expressing either wt or mutUGV1-NP-FLAG, UHV1-NP-FLAG, or HISV1-NP-FLAG **(a)**, and wt or mutJUNV-NP-HA, or LCMV-NP-HA **(b)** at three dpt, after incubation at 30°C. Non-transfected (Mock) cells served as controls. **(a)** The panels from left: FLAG tag in red (secondary antibody: AlexaFluor 594 goat anti-rabbit), mitochondrial marker in green (secondary antibody: AlexaFluor 488 goat

anti-mouse), nuclei in blue (DAPI), and a merged image. **(b)** The panels from left: HA tag in red (secondary antibody: AlexaFluor 594 goat anti-rabbit), mitochondrial marker in green (secondary antibody: AlexaFluor 488 goat anti-mouse), nuclei in blue (DAPI), and a merged image. Some of the NP inclusions/aggregates present in cells expressing either wtUGV1-NP-FLAG, wt or mutJUNV-NP-HA are indicated by white arrowheads.

Supplementary Figure S11

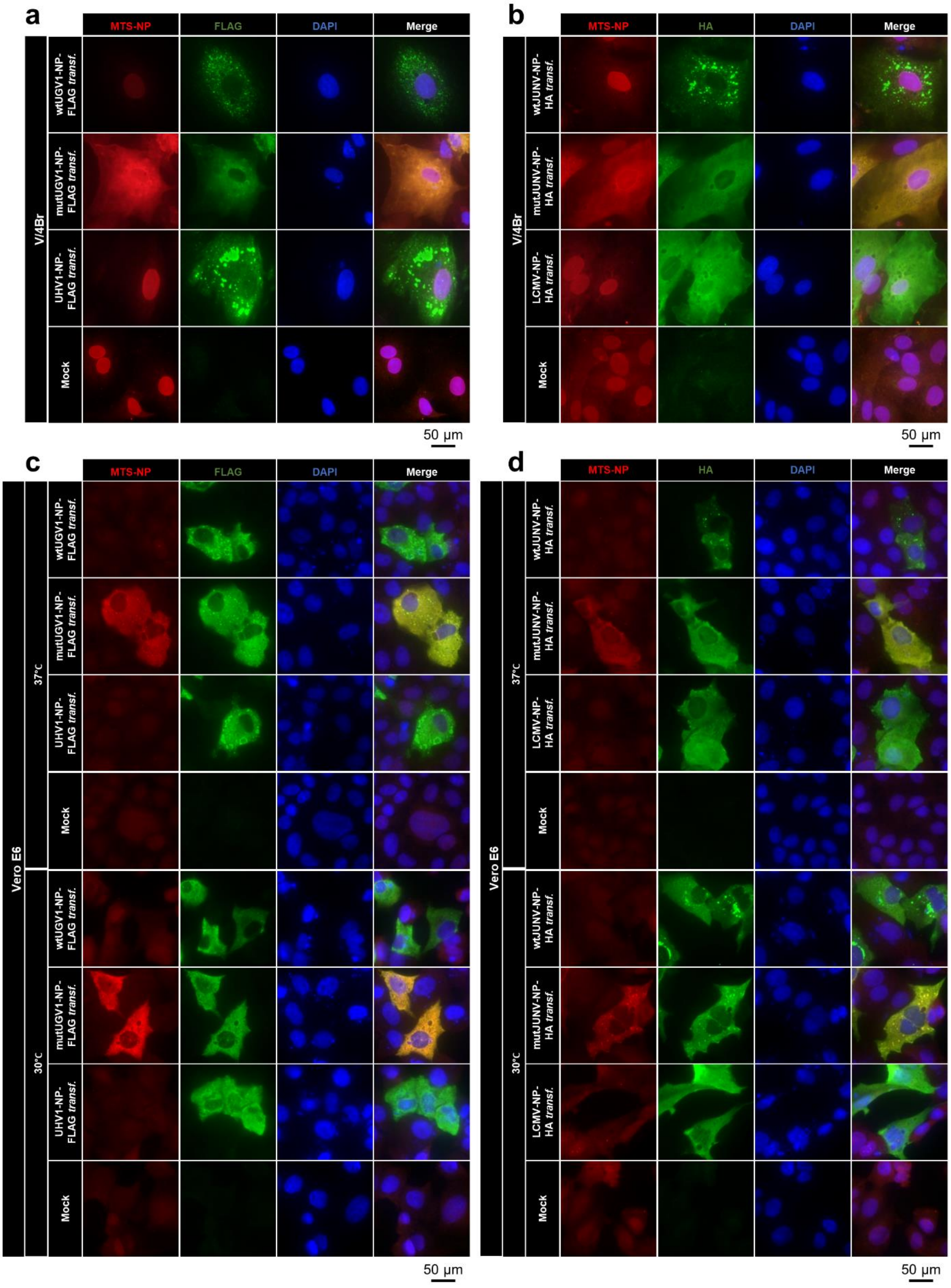

Supplementary Figure S11: IF studies on the putative MTS of arenaviral NPs in transfected boid or mammalian cells. (a, b) Double IF images of *Boa constrictor* V/4Br cells

transfected with a construct expressing either wt or mutUGV1-NP-FLAG, or UHV1-NP-FLAG **(a)**, and wt or mutJUNV-NP-HA, or LCMV-NP-HA **(b)** at three dpt. **(c, d)** Double IF images of monkey Vero E6 cells transfected with a construct expressing either wt or mutUGV1-NP-FLAG, or UHV1-NP-FLAG **(c)**, and wt or mutJUNV-NP-HA, or LCMV-NP-HA **(d)** at three dpt after incubation at either 37°C (upper side) or 30°C (lower side). Non-transfected cells (Mock) served as controls. **(a, c)** The panels from left: MTS-NP in red (secondary antibody: AlexaFluor 594 goat anti-rabbit), FLAG tag in green (secondary antibody: AlexaFluor 488 goat anti-mouse), nuclei in blue (DAPI), and a merged image. **(b, d)** The panels from left: MTS-NP in red (secondary antibody: AlexaFluor 594 goat anti-rabbit), HA tag in green (secondary antibody: AlexaFluor 488 goat anti-mouse), nuclei in blue (DAPI), and a merged image.

Supplementary Figure S12

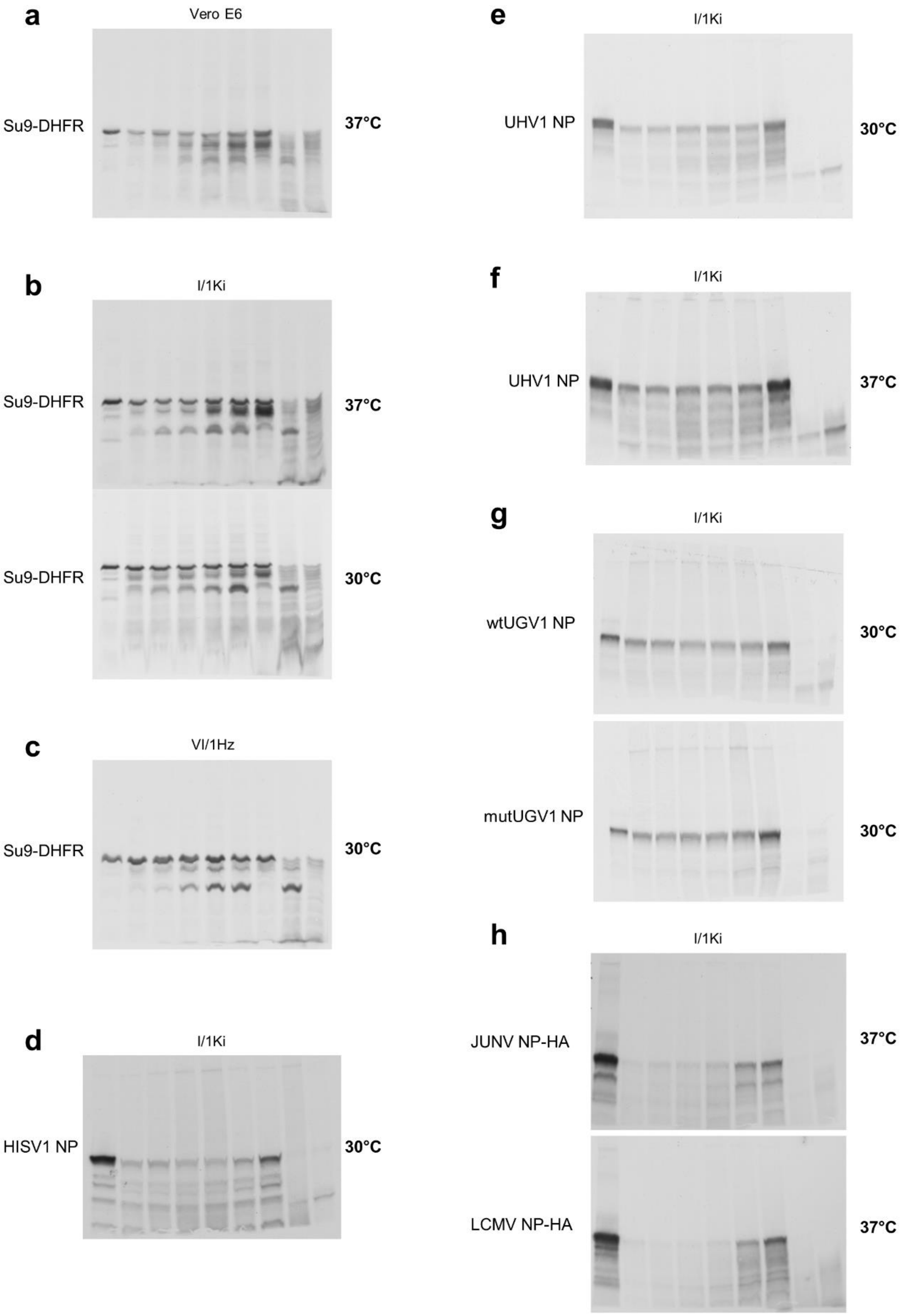

Supplementary Figure S12: Full-length autoradiographies of *in vitro* mitochondria import assay of arenaviral NPs. Full-length/uncropped versions of the autoradiographies of *in vitro* import into mitochondria assay presented in Fig. 7, panels a-h. **(a-c)** The known chimeric mitochondrial protein, Su9-DHFR, used as positive control, was imported into freshly isolated mitochondria of monkey Vero E6 cells, at 37°C **(a)**, *Boa constrictor* kidney (I/1Ki) cells, at both 37°C and 30°C **(b)**, and *Python regius* heart (VI/1Hz) cells, at 30°C **(c)**. **(d-h)** *In vitro* translocation into freshly isolated *Boa constrictor* I/1Ki mitochondria assessed for HISV-1 NP, at 30°C **(d)**, UHV-1 NP, at 30°C **(e)** and 37°C **(f)**, wt and mutUGV-1 NPs, at 30°C **(g)** and HA-tagged JUNV and LCMV NPs, at 37°C **(h)**. Radiolabelled Su9-DHFR and NPs were *in vitro* synthesized using [^{35}S]-methionine in a rabbit reticulocyte lysate. Protein signals were determined through autoradiographic detection.

Supplementary Figure S13

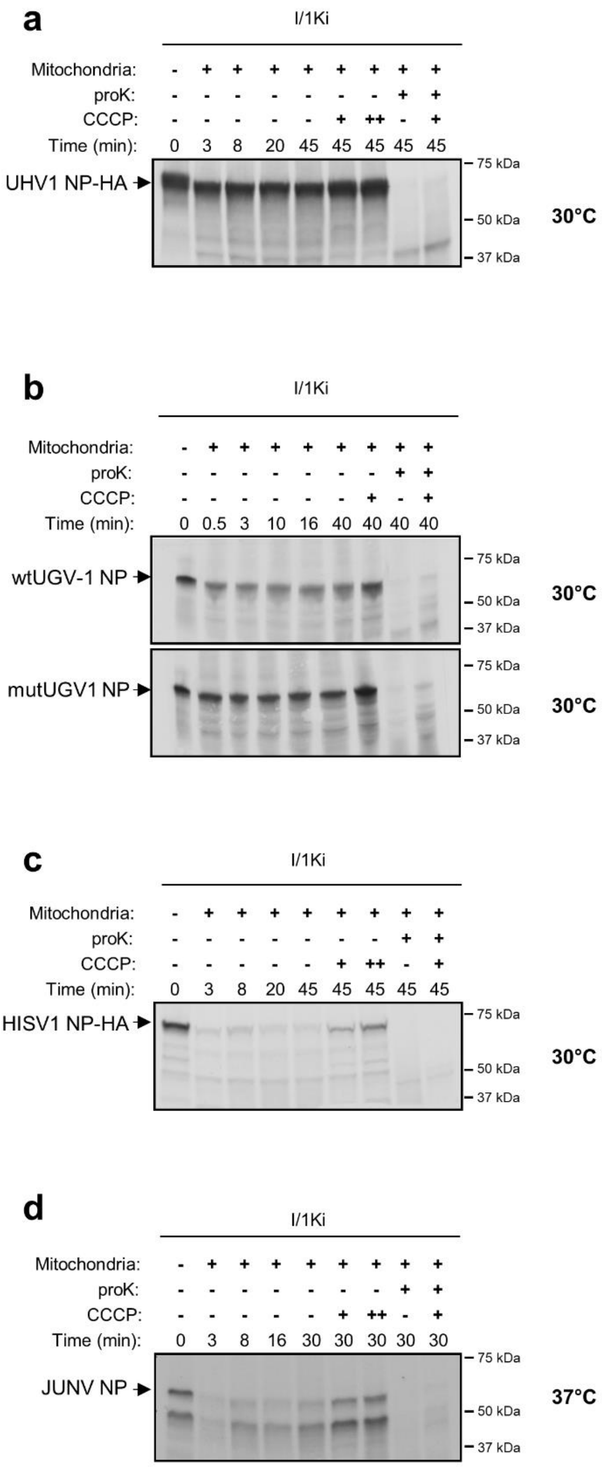

Supplementary Figure S13: Analyses of *in vitro* import into mitochondria of arenavirus NPs. (a-d) The *in vitro* translocation into freshly isolated *Boa constrictor* I/1Ki mitochondria was assessed for HA-tagged UHV-1 NP, at 30°C **(a)**, wt and mut UGV-1 NPs, at 30°C **(b)**, HA-

tagged HISV-1 NP, at 30°C **(c)** and JUNV NP, at 37°C **(d)**. Radiolabelled NPs were *in vitro* synthesized using [^{35}S]-methionine in a rabbit reticulocyte lysate, from their ORFs cloned into pCR4Blunt-TOPO **(a, b)** or pGEM4Z **(c, d)** vectors, flanking the T7 **(a-c)** or SP6 **(d)** promoter. Protein signals were determined through autoradiographic detection. CCCP: mitochondrial protein import blocker by inducing mitochondrial membrane potential dissipation. proK: leading to degradation of non-imported proteins. Full-length autoradiographies are presented in Supplementary Figure S14.

Supplementary Figure S14

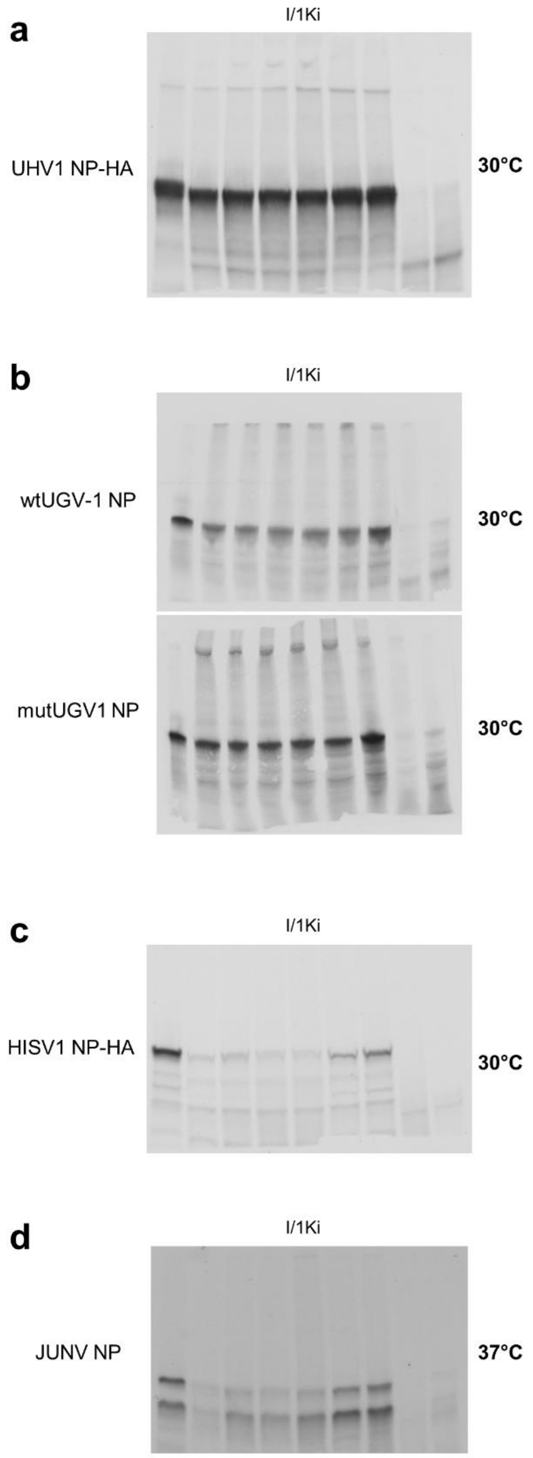

Supplementary Figure S14: Full-length autoradiographies of analyses on *in vitro* mitochondria import of arenaviral NPs. (a-d) Full-length/uncropped versions of the autoradiographies of *in vitro* mitochondria import assay presented in Supplementary Fig. S13, panels a-d, assessed on freshly isolated *Boa constrictor* I/1Ki mitochondria for HA-tagged

UHV-1 NP, at 30°C **(a)**, wt and mut UGV-1 NPs, at 30°C **(b)**, HA-tagged HISV-1 NP, at 30°C **(c)** and JUNV NP, at 37°C **(d)**. Radiolabelled NPs were *in vitro* synthesized using [^{35}S]-methionine in a rabbit reticulocyte lysate. Protein signals were determined through autoradiographic detection.
